# Gray matter hypoperfusion is a late pathological event in the course of Alzheimer’s disease

**DOI:** 10.1177/0271678X221141139

**Published:** 2022-11-22

**Authors:** Khazar Ahmadi, Joana B Pereira, David Berron, Jacob Vogel, Silvia Ingala, Olof T Strandberg, Shorena Janelidze, Frederik Barkhof, Josef Pfeuffer, Linda Knutsson, Danielle van Westen, Sebastian Palmqvist, Henk JMM Mutsaerts, Oskar Hansson

**Affiliations:** 1 Clinical Memory Research Unit, Department of Clinical Sciences, Lund University, Lund, Sweden; 2 Institute of Cognitive Neuroscience, Faculty of Psychology, Ruhr University Bochum, Bochum, Germany; 3 Division of Clinical Geriatrics, Department of Neurobiology, Care Sciences and Society, Karolinska Institute, Stockholm, Sweden; 4 Department of Psychiatry, University of Pennsylvania, Philadelphia, Pennsylvania, USA; 5 Department of Radiology and Nuclear Medicine, Amsterdam University Medical Center, Location VUmc, Amsterdam Neuroscience, Amsterdam, the Netherlands; 6 Queen’s Square Institute of Neurology and Centre for Medical Image Computing, University College London, London, UK; 7 Application Development, Siemens Healthcare, Erlangen, Germany; 8 Department of Medical Radiation Physics, Lund University, Lund, Sweden; 9 The Russell H. Morgan Department of Radiology and Radiological Science, Division of MR Research, The Johns Hopkins University School of Medicine, Baltimore, MD, USA; 10 Diagnostic Radiology, Lund University, Lund, Sweden; 11 Department of Neurology, Skåne University Hospital, Lund, Sweden; 12 Memory Clinic, Skåne University Hospital, Malmö, Sweden

**Keywords:** Alzheimer’s disease, arterial spin labeling, cerebral blood flow, neurodegeneration, tau

## Abstract

Several studies have shown decreased cerebral blood flow (CBF) in Alzheimer’s disease (AD). However, the role of hypoperfusion in the disease pathogenesis remains unclear. Combining arterial spin labeling MRI, PET, and CSF biomarkers, we investigated the associations between gray matter (GM)-CBF and the key mechanisms in AD including amyloid-β (Aβ) and tau pathology, synaptic and axonal degeneration. Further, we applied a disease progression modeling to characterize the temporal sequence of different AD biomarkers. Lower perfusion was observed in temporo-occipito-parietal cortex in the Aβ-positive cognitively impaired compared to both Aβ-negative and Aβ-positive cognitively unimpaired individuals. In participants along the AD spectrum, GM-CBF was associated with tau, synaptic and axonal dysfunction, but not Aβ in similar cortical regions. Axonal degeneration was further associated with hypoperfusion in cognitively unimpaired individuals. Disease progression modeling revealed that GM-CBF disruption Followed the abnormality of biomarkers of Aβ, tau and brain atrophy. These findings indicate that tau tangles and neurodegeneration are more closely connected with GM-CBF changes than Aβ pathology. Although subjected to the sensitivity of the employed neuroimaging techniques and the modeling approach, these findings suggest that hypoperfusion might not be an early event associated with the build-up of Aβ in preclinical phase of AD.

## Introduction

Alzheimer’s disease (AD) is a neurodegenerative disorder characterized by the accumulation of amyloid-β (Aβ) plaques and neurofibrillary tangles of hyperphosphorylated tau. These pathological changes precede the onset of dementia by many years, leading to the conceptualization of AD as a continuum evolving from preclinical to prodromal stages and finally to dementia.^1,2^ Multiple neuroimaging studies have shown alterations in functional connectivity,^3–5^ brain metabolism^6–8^ and perfusion^9,10^ in different cortical regions through the course of AD. The latter can be noninvasively measured with arterial spin labeling (ASL) MRI which utilizes magnetically labeled water in the blood as an endogenous tracer.^11^ Due to the close coupling of glucose metabolism and cerebral blood flow (CBF), the patterns of regional hypometabolism on well-established positron emission tomography with [18F]-fluorodeoxyglucose (FDG-PET) overlap with reduced CBF on ASL scans.^7,12^ This renders ASL as a promising radiation-free candidate for the diagnosis and monitoring of AD.

Despite several reports on CBF changes in AD,^13–15^ their association with the core pathologies including Aβ and tau burden remains elusive. In a previous study, a negative correlation was reported between amyloid-PET and ASL-CBF in temporo-parietal regions across the AD continuum.^10^ In contrast, other studies have suggested a link between cerebrospinal fluid (CSF) biomarkers of tau, but not Aβ, with perfusion abnormalities on single photon emission computed tomography.^16,17^ The few available studies on the relationship between ASL-CBF and tau-PET have yielded mixed findings. While one study has found negative associations in widespread temporo-parietal regions,^18^ another one has shown that higher tau-PET uptake is correlated with hypoperfusion exclusively in the entorhinal cortex.^19^ Moreover, no studies to date have investigated the associations of ASL-CBF with synaptic and axonal markers in the CSF.

There is also the important question whether decreased CBF triggers the chain of pathological events in AD, or if it is a consequence of the downstream effects of Aβ and tau. Thus, the overarching aims of the current study were to examine the multimodal relationships between ASL-CBF with distinct PET/CSF biomarkers and global cognition across the AD spectrum and to identify when along the disease evolution ASL-CBF changes occur. To this end, we first measured ASL-CBF variations in Aβ-positive or negative cognitively unimpaired individuals and cognitively impaired patients with Aβ pathology. We then evaluated the association between CBF and deposition of Aβ and tau measured with amyloid-PET and CSF Aβ42/40, tau-PET and CSF phosopho-tau217 (P-tau217), as well as the CSF concentrations of synaptic (Neuronal Pentraxin2 to total tau ratio; NPTX2/T-tau), and axonal (neurofilament light chain; NfL) biomarkers. Finally, we ran a linear event progression modeling^20^ approach to estimate the order of abnormality of these markers. Specifically, we addressed the following questions. i) When does CBF change in the AD continuum? ii) Which brain regions are primarily affected in AD by CBF alterations? iii) Are CBF changes associated with Aβ or tau burden in the AD spectrum? iv) Are CBF variations corelated with markers of synaptic or axonal integrity? Our results suggest that CBF alterations occur at later stages of the AD continuum. These changes are most pronounced in the temporo-occipito-parietal areas and are mainly associated with tau load, synaptic and axonal degeneration.

## Methods

### Participants

**Figure 1.**
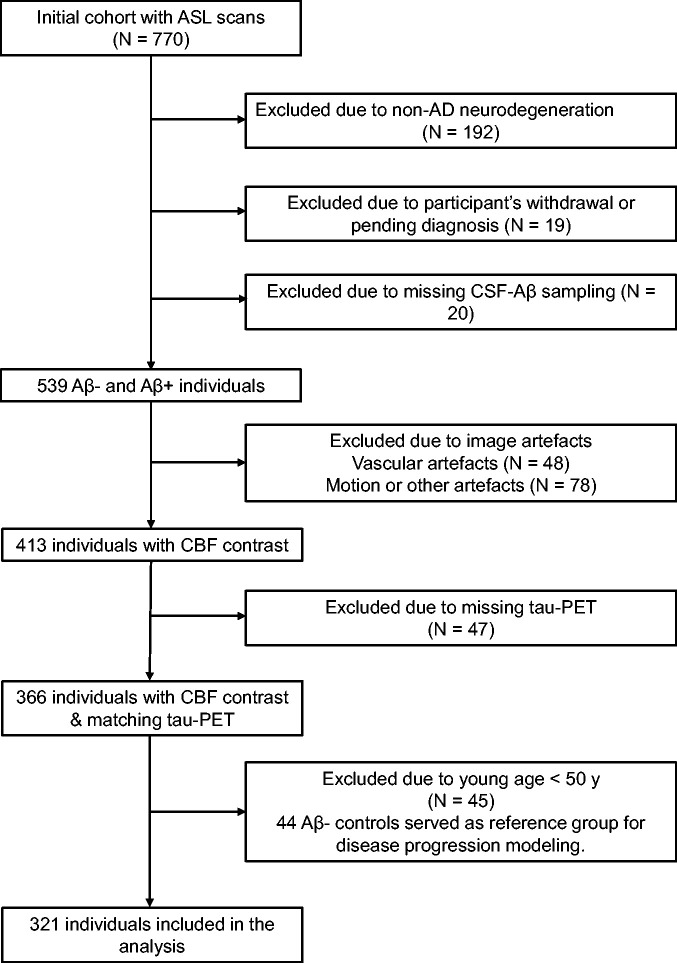
Inclusion/exclusion pathway. Flowchart shows the initial participant population with ASL scans and those excluded due to non-AD neurodegeneration, missing CSF sampling, participant’s withdrawal, vascular or other sources of artefacts e.g., excessive motion or poor background suppression, missing tau-PET data and young age. The non-AD group comprised the following diagnoses: behavioral variant fronto-temporal dementia (N = 10), Parkinson’s disease with dementia (N = 4), vascular dementia (N = 10), dementia with Lewy bodies (N = 24), Parkinson’s disease^22^ (N = 33), progressive supranuclear palsy^23^ (N = 17), multiple system atrophy^24^ (N = 7), corticobasal syndrome^25^ (N = 2), semantic variant primary progressive aphasia and primary non-fluent aphasia^26^ (N = 5 and 1, respectively), Aβ-negative mild cognitive impairment (N = 60) and unspecified dementia (N = 19). The diagnoses were made by neurologists following clinical and neuropsychological evaluation fulfilling the respective criteria based on Diagnostic and Statistical Manual of Mental Disorders (DSM-5) or the corresponding above-mentioned references. Note that of 45 individuals excluded due to the young age, one was positive for Aβ pathology and therefore was not included in the reference group for disease progression modeling.

The participants were recruited from the ongoing Swedish BioFINDER-2 study ( NCT03174938 ), which has been previously described in detail.^21^ The study comprised cognitively unimpaired (CU) controls, and cognitively impaired (CI) patients diagnosed either with mild cognitive impairment (MCI) or AD with dementia. The groups were further stratified into Aβ-positive and negative individuals based on the CSF Aβ42/40 ratio. Of 770 individuals with ASL scans, 321 individuals were included in the main analysis (see Figure 1). A subset of participants had taken part in a previous study to identify discriminative accuracy of plasma P-tau217 in AD vs non-AD disorders^21^ and thereby had available PET, structural MRI and CSF biomarkers of Aβ42/40 and P-tau217 (see Supplementary Table 1 for quantification of overlapping participants between the two studies). Despite the overlap in participants’ inclusion, there is no overlapping results between the two studies. Demographic and clinical characteristics of the participants are summarized in Table 1. All participants gave written informed consent. The study was in accordance with the Declaration of Helsinki and was approved by the Ethical Review Board in Lund, Sweden.

**Table 1.** Demographic summary.

	Amyloid-negative CU (N = 142)	Amyloid-positive CU (N = 60)	Amyloid-positive CI (N = 119)	p value
Males/females	64/78	26/34	49/70	<0.0001
Age	65.51 (10.45)	71.69 (8.37)	72.90 (6.86)	<0.0001
Years of education	12.58 (3.67)	12.93 (4.04)	12.71 (4.44)	0.782
APOE ε4 carriers%	32.39%	71.66%	73.94%	<0.0001
MMSE	28.90 (1.18)	28.61 (1.46)	23.66 (4.39)	<0.0001

Data are presented as mean values followed by (standard deviations). Demographic factors were compared between groups using X^2^ and ANOVA tests. MMSE: Mini-Mental State Examination. Out of 119 CI patients, 55 had MCI and 64 had AD with dementia.

### MRI acquisition and processing

ASL scans were acquired using a prototype 3D pseudo-continuous (pCASL) sequence with background suppression and gradient- and spin-echo (GRASE) readout in a 3T MAGNETOM Prisma scanner with a 64-channel receiver-coil array (Siemens Healthcare, Erlangen, Germany). The labeling plane was planned using an MR-angiogram and the following parameters were used for the pCASL sequence: TR|TE = 4600|21.76 ms, flip angles (FA) of the sequence excitation/refocusing and labeling RF pulse = 90°/180° and 28° respectively, labeling duration|post-labeling delay =1500|2000 ms, interpolated voxel size = 1.9 ×1.9 × 4 mm^3^ (initial acquisition resolution = 3.75 × 3.75 × 4 mm^3^), FoV = 240 mm, GRAPPA factor = 2, Turbo factor = 12, EPI factor = 31, 3 segments, 36 axial slices, 12 pairs of control/label images and total acquisition time of 5:50 min. A proton density weighted (M0) volume with TR = 4000 ms was also acquired to calibrate estimated CBF into absolute units. Halfway through the study, the ASL prototype sequence was updated. The protocol was adjusted to optimally resemble the pre-update version. The post-update version included changes in phase encoding direction (RL to AP), coil combine mode (‘sum of squares’ to ‘adaptive combine’) resulting in an improved signal-to-noise-ratio (SNR), TE reduction from 21.76 to 20.76 ms, an increase in TR of the M0 image by 1 second (4000 to 5000 ms) and an unbalanced vs balanced labeling scheme. There were no between-group differences in demographics pre- and post-update (Supplementary Table 2). However, estimated global GM-CBF was higher in those scanned after the sequence update (see Supplementary Figure 1), potentially explained by changes in the labeling scheme. The ASL sequence version was included as a covariate in all the subsequent regression analysis.^27^

Additionally, a whole-brain T1-weighted scan (MPRAGE sequence, TR|TE|TI = 1900|2.54|900 ms, resolution = 1 × 1 × 1 mm^3^, FA = 9°, FoV = 256 ×256 mm^2^, 176 slices, bandwidth = 220 Hz/px TA =5:15 min) and a T2-weighted FLAIR scan (TR|TE|TI = 5000|393|1800 ms, bandwidth = 781 Hz/px TA =4:37 min, same resolution and FoV as for the T1-weighted image) were obtained. ASL and structural scans were processed using the publicly available ExploreASL toolbox (https://github.com/ExploreASL/ExploreASL) developed in MATLAB which uses the Statistical Parametric Mapping (SPM 12) routines. The processing pipeline has been described in detail elsewhere.^28^ Briefly, white matter hyperintensities (WMH) were segmented on the FLAIR image and used to fill the corresponding hypointensities on T1-weighted scan. Voxel intensities in these lesion regions were replaced by bias-field corrected values from the surrounding normally appearing WM on T1 image. Subsequently, the anatomical image was segmented into gray matter (GM), WM and CSF using computational anatomy toolbox 12 (CAT12) to obtain partial volume (PV) maps which were normalized to the MNI152 space. ASL scans were motion-corrected and co-registered to the T1 scan. The M0 image was smoothed with a 16 mm FWHM Gaussian kernel. CBF was quantified using a single compartment model^29^ as follows:

$$\mathrm{CBF}=\frac{6000*\lambda *\left(\mathrm{control}-\mathrm{label}\right)*e^{\mathrm{PLD}/T1a}}{2*\alpha *T1a*M0*(1-e^{-\tau /T1a})}[\text{m}\text{l}/100\text{g}/\text{min}]$$

where λ is the brain/blood partition coefficient (0.9 ml/g), PLD is post-labeling delay (2000 ms), T1a is the longitudinal relaxation time of arterial blood, α is the labeling efficiency (85%), M0 is the proton density image intensity and τ is the labeling duration (1500 ms). To distinguish perfusion changes from concomitant cerebral atrophy, voxel-wise PV effects were corrected and transformed to the ASL image space in a linear regression algorithm using a 3D Gaussian kernel. Subsequently, the CBF images were transformed into MNI152 space. Finally, ASL scans were quality controlled by an automatic method which ranks the processed images based on their spatial coefficient of variation (sCoV), reflecting the amount of arterial transit time (ATT) artefacts.^30^ This procedure was reviewed by a trained radiologist (S.I., with 5 years of experience) to detect vascular or other sources of artefacts. Exemplary ASL scans with CBF contrast and excluded images are presented in Supplementary Figure 2. Region of interest (ROI) masks were obtained by intersecting the Harvard-Oxford (HO) atlas with individual GM and WM masks. Mean bilateral PV-corrected GM-CBF values were calculated for the atlas ROIs in the standard space.^28^

### Regions of interest definition for CBF

**Figure 2.**
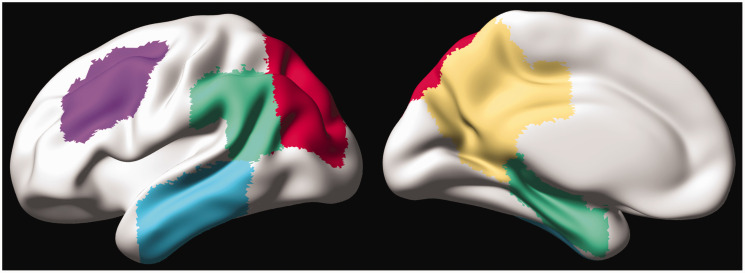
A priori-defined CBF-ROIs projected on the cortical surface. The left and right panels represent the lateral and medial views, respectively. The ROIs are displayed with distinct colors (lateral temporal = cyan, lateral parietal = green, superior lateral occipital = red, middle frontal = violet, medial parietal = yellow, and medial temporal = green).

In line with previous studies, regional GM-CBF values for the atlas ROIs were normalized to CBF of the precentral gyrus to adjust for individual variations in the blood flow.^10,31^ No between group CBF differences were found in this region, prior to the normalization. A set of *a priori* ROIs were defined based on previous studies showing CBF alterations within these regions in the AD spectrum. These *a priori* defined ROIs encompassed lateral and medial parts of the parietal and temporal cortex, middle frontal gyrus and superior division of the lateral occipital cortex (Figure 2). The lateral and medial parietal meta-ROIs consisted of anterior/posterior supramarginal gyri, angular gyrus and posterior cingulate plus precuneus, respectively. The lateral temporal meta-ROI included anterior/posterior divisions of the middle and inferior temporal gyri while hippocampus and anterior/posterior divisions of parahippocampal gyrus (anterior parahippocampal gyrus in HO atlas represents the entorhinal cortex) were grouped into the medial temporal meta-ROI. The weighted-mean CBF for a *priori* ROIs was obtained by the sum of products of the mean GM-CBF in each sub-ROI and its volume, divided by the sum of the volumes of all the sub-ROIs constituting each *priori* ROI.

### PET acquisition and processing

Tau-PET scans using [18F] RO948 were performed on a GE Discovery MI scanner (General Electric Medical Systems) 70–90 minutes post-injection. [18F] flutemetamol Aβ-PET scans were acquired on the same scanner 90–110 minutes following the injection as previously described.^32^ Standardized uptake value ratio (SUVR) images were calculated using the inferior cerebellar GM and pons as the reference regions for tau- and Aβ-PET, respectively. The aforementioned T1-weighted scan was used for PET image co-registration and template normalization. Note that the Aβ-PET data were only available for a subset of 245 participants, including 132 Aβ-negative, 57 Aβ-positive CU individuals and 56 Aβ-positive CI patients.

To cover brain regions affected by tau and Aβ pathology, composite-ROIs were created using the Freesurfer-based anatomical parcellation (https://surfer.nmr.mgh.harvard.edu/). Tau meta-ROIs (illustrated in Supplementary Figure 3) approximated Braak staging scheme^33^ and included an early (entorhinal) ROI corresponding to Braak stage I–II, a temporal meta-ROI reflecting Braak stage I–IV, and a late neocortical meta-ROI corresponding to Braak stage V–VI.^34^ The amyloid-β neocortical composite ROI encompassed prefrontal, parietal, temporal lateral, anterior/posterior cingulate, and precuneus.^35,36^ The overall distributions of Aβ- and tau-PET as well as ASL-CBF scans in four representative subjects are shown in Supplementary Figure 4.

### CSF biomarkers

Lumbar CSF sampling and analysis were conducted according to the Alzheimer’s Association Flow Chart.^37^ As previously described, the analysis of P-tau217 assays was performed using the Meso Scale Discovery (MSD) platform.^38^ Total tau (T-tau) was quantified using Innotest® immunoassay (Fujirebio; Gent, Belgium). CSF Aβ42 and Aβ40 levels were measured using MSD or Fujirebio Lumipulse assays according to the manufacturer’s instructions, and the ratio values were subsequently calculated. NfL was measured using a Simoa kit (Quanterix; Billerica, MA). NPTX2 was measured in the facilities of ADx (Gent, Belgium) using a research grade sandwich ELISA. The number of available data per group for each of the CSF and imaging biomarkers together with the corresponding mean ± SD values are provided in Supplementary Table 3.

### Statistical analyses

The ROI-based analyses were performed using R (version 3.5.2). Group differences across demographics were compared using analysis of variance (ANOVA) or chi-squared tests (Table 1). CBF differences between the diagnostic groups were evaluated by univariate linear regression models with GM-CBF of *a priori* ROIs as the dependent variable and the groups as the independent variable. To assess the relationships between CBF and each of the PET and CSF markers of Aβ, tau, synaptic dysfunction and axonal integrity, the regression analysis was separately applied to the AD continuum i.e., those with Aβ pathology regardless of their cognitive status and CU participants with the GM-CBF in a *priori* ROIs as the dependent variable and each biomarker as the independent variable. All the analyses were controlled for the confounding effects of age, sex and ASL sequence version. The distribution of NfL was skewed and required a logarithmic transformation. The results were considered statistically significant at p < 0.05. False discovery rate (FDR) correction was used to adjust for multiple comparisons.

### Voxel-wise analyses

In addition to the ROI-based analyses, whole-brain voxel-wise regression analyses were performed to compare GM-CBF variations between groups and to assess the spatial associations of CBF with Aβ, tau and other CSF markers in both CU individuals and those on the AD spectrum. All voxel-wise regression analyses were controlled for age, sex and ASL sequence version. The CBF images were multiplied by a GM mask and subsequently smoothed with a Gaussian kernel of 4 mm FWHM. The analyses were performed using threshold-free, cluster-enhanced (TFCE) permutation statistics using FSL randomise command with 5000 permutations (https://fsl.fmrib.ox.ac.uk/fsl/fslwiki/Randomise). Statistical significance was set at the family-wise error (FWE) corrected threshold of p < 0.05.

### Disease progression modeling

A linear event progression model^20^ was employed to characterize the progression of AD in terms of different biomarkers via the pySuStaIn package^39^ (https://github.com/ucl-pond/pySuStaIn). This approach builds on event-based modeling^40,41^ delineating the disease progression as a series of events, each of which corresponds to a biomarker switching from a normal to an abnormal level. It replaces the instantaneous normal to abnormal transition of events with a linear z-score model in which the trajectory of each subtype is described by linear accumulation of a biomarker from one z-score to another over different stages.^20^ Markov Chain Monte Carlo sampling is then used to quantify the uncertainty of the sequence ordering upon which a positional variance diagram can be visualized.^20^ Seven biomarkers that are most commonly associated with AD were fed into the linear event progression model. They comprised CSF levels of Aβ42/40 and P-tau217, tau-PET deposition in early, temporal/limbic and late ROIs, a composite volumetric measure in AD signature regions i.e., bilateral entorhinal, inferior/middle temporal and fusiform cortices^34,42^ and weighted mean GM-CBF of regions having voxel-wise associations with tau-PET uptake in the early ROI (Figure 4(a)) in the AD spectrum. Note that in the Aβ-negative CU individuals, the GM-CBF values were extracted based on the same mask of significant voxel-wise associations in the AD spectrum (see Figure 4(a)). We chose to derive mean CBF from these regions to reflect on areas undergoing early changes as tau accumulation in the entorhinal cortex is among the initial changes occurring during the AD development. To ensure that the temporal ordering of the GM-CBF changes is not influenced by the ROI choice, we repeated the analysis by also estimating i) mean GM-CBF of all a *priori* defined ROIs (Figure 2), ii) weighted mean GM-CBF in two medial parietal ROIs including precuneus and posterior cingulate as the main regions for early amyloid accumulation and iii) global GM-CBF.

The values for each biomarker were represented as a z-score relative to Aβ-negative CU subjects who were younger than 50 years old (N = 44, see Figure 1), and event thresholds were set at 1, 2 and 3 standard deviations from the young healthy controls. Except for being a reference (control population) for the model, these data were not included in any other analyses (Figure 1). Participants with at least one missing value in any of the aforementioned markers were excluded. As such, the model was run on a sample of 259 individuals, a ratio of ∼12 subjects per feature (7 biomarkers × 3 waypoints). This ratio is in line with previous work using the same algorithm,^43^ and benchmarking a similar algorithm suggests this ratio is sufficient for accurately recovering the true sequence of simulated data.^44^ Furthermore, the linear event progression model provides internal measures of uncertainty, allowing an approximation of confidence in different stages of the event sequence.

### Data availability

Anonymized data will be shared by request from a qualified academic investigator for the sole purpose of replicating procedures and results presented in the article and as long as data transfer is in agreement with EU legislation on the general data protection regulation and decisions by the Ethical Review Board of Sweden and Region Skåne, which should be regulated in a material transfer agreement.

## Results

### GM-CBF differences between diagnostic groups

#### Aβ-negative CU vs. Aβ-positive CU

No significant difference in GM-CBF was observed when comparing Aβ-negative and Aβ-positive CU individuals, neither with the ROI-based nor with the voxel-wise analyses (see Supplementary Table 4).

#### Aβ-negative CU vs. Aβ-positive CI

**Figure 3.**
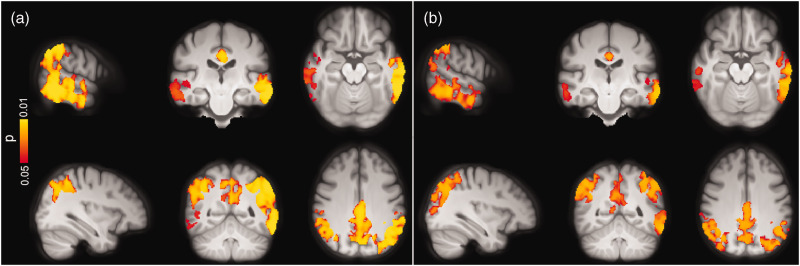
Voxel-wise group differences between (a) Aβ-negative CU vs. Aβ-positive CI and (b) Aβ-positive CU vs. Aβ-positive CI individuals adjusted for age, sex and ASL sequence version. Clusters with significantly decreased GM-CBF in Aβ-positive CI patients are overlaid on the mean population T1-weighted volume in radiological space (p < 0.05, FWE corrected). The top and bottom panels display different representative slices.

The ROI-based analyses revealed significant GM-CBF reductions in the lateral parietal (β = −0.041, p_(FDR)_ = 0.0042), lateral temporal (β = −0.046, p_(FDR)_ = 0.0035), superior lateral occipital (β = −0.059, p_(FDR)_ = 0.0035) and middle frontal (β = −0.058, p_(FDR)_ = 0.0012) ROIs in Aβ-positive CI individuals compared to Aβ-negative CU participants. These findings were further confirmed by the voxel-wise analyses (Figure 3(a)), demonstrating decreased GM-CBF in the bilateral superior lateral occipital, bilateral lateral/medial parietal, and the left lateral temporal regions (p < 0.05, FWE-corrected).

#### Aβ-positive CU vs. Aβ-positive CI

The overall hypoperfusion pattern in Aβ-positive CI with respect to Aβ-positive CU individuals was similar to the pattern observed in the same group when compared to Aβ-negative CU subjects, albeit less pronounced. Specifically, lower GM-CBF was observed in the superior lateral occipital (β = −0.081, p_(FDR)_ = 0.0028) and middle frontal (β = −0.061, P_FDR_ = 0.0033) ROIs in Aβ-positive CI individuals. As depicted in Figure 3(b), the voxel-wise analyses showed decreased GM-CBF in the lateral temporal, medial/lateral parietal and superior lateral occipital cortices, largely in line with the ROI-based analyses (p < 0.05, FWE-corrected).

Additional correction for the APOE ε4 status did not alter the observed between-group findings (see Supplementary Table 5 and Supplementary Figure 5).

### Correlations between GM-CBF and Aβ pathology

Next, we studied the associations between CBF and Aβ pathology measured by Aβ-PET SUVR in the neocortical composite ROI and CSF levels of Aβ42/40. In CU individuals, no associations were observed between CBF and markers of Aβ, neither with the ROI-based nor with the voxel-wise analyses. Similarly, CBF was not correlated with Aβ pathology in individuals along the AD continuum (Supplementary Table 6).

### Correlations between GM-CBF and tau pathology

**Figure 4.**
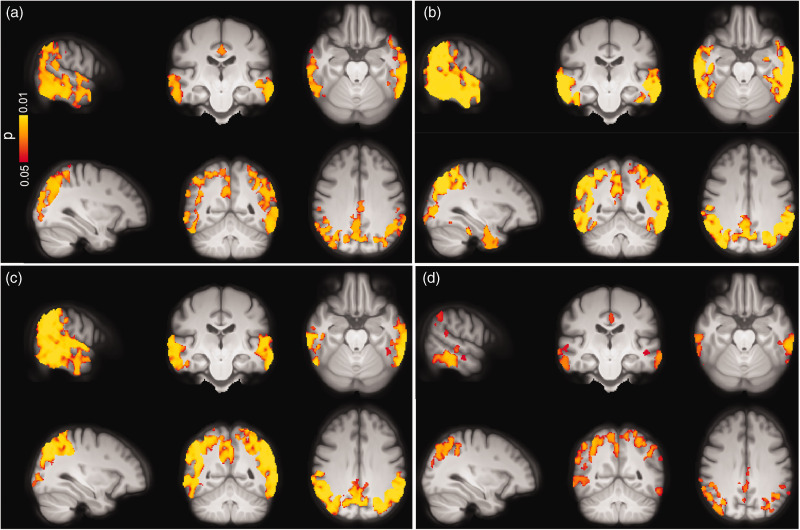
Voxel-wise associations between GM-CBF and tau uptake in (a) early, (b) temporal, (c) late ROIs and (d) CSF concentrations of P-tau217 in the AD spectrum adjusted for age, sex and ASL sequence version. CBF reduction was primarily observed in lateral temporal, lateral/medial parietal and superior lateral occipital regions although its spatial extent was moderately attenuated for P-tau217 compared to tau-PET (p < 0.05, FWE corrected). Conventions as for Figure 3.

To study associations between CBF and tau pathology, we initially used the tau-PET data. No significant associations were found between CBF and tau load in any of the early, temporal and late tau-PET ROIs in CU individuals regardless of Aβ status. Additionally, GM-CBF was not associated with CSF levels of P-tau217 in this group (see Supplementary Figure 6). In individuals on the AD spectrum, however, both the ROI-based and voxel-wise analyses demonstrated inverse associations between GM-CBF and tau biomarkers. Higher tau-PET uptake in the early ROI was significantly associated with decreased GM-CBF in the lateral parietal (β = −0.048, p_(FDR)_ = 0.0117), lateral temporal (β = −0.052, p_(FDR)_ = 0.0117), superior lateral occipital (β = −0.084, p_(FDR)_ = 0.0025) and middle frontal (β = −0.045, p_(FDR)_ = 0.0273) ROIs. Moreover, GM-CBF in the same regions were negatively associated with tau burden in the temporal and late meta-ROIs, respectively [lateral parietal (β = −0.056|−0.119, p_(FDR)_ = 0.00008|0.00002), lateral temporal (β = −0.062|−0.086, p_(FDR)_ = 0.00005|0.00217), superior lateral occipital (β = −0.089| 0.126, p_(FDR)_ = 0.00002|0.00092) and middle frontal (β = −0.044|−0.101, p_(FDR)_ = 0.00682|0.00092)]. The associations of CBF with tau-PET ROIs were still significant after controlling for CSF concentrations of Aβ42/40 (Supplementary Table 7). Analogous findings were observed after controlling for cognitive status and the APOE ε4 status (see Supplementary Table 8 and Supplementary Figure 7). The overall significant associations between GM-CBF and tau uptake in each of the three ROIs are plotted in Supplementary Figure 8. Further using the voxel-wise analyses, similar patterns of hypoperfusion were observed for all three tau-PET ROIs mainly in the temporo-occipito-parietal regions (Figure 4(a) to (c)). Except for the tau-PET SUVRs in the early ROI, additional adjustment for the cognitive status did not alter the observed voxel-wise associations, as shown in Supplementary Figure 9. This also held true when controlling for CSF levels of Aβ42/40 and APOE ε4 status (see Supplementary Figure 10 and Supplementary Figure 11 A-C, respectively).

Afterwards, we examined the associations between GM-CBF and CSF P-tau217 which is an earlier marker than tau PET.^45^ Elevated CSF levels of P-tau217 were correlated with lower CBF in lateral parietal (β =−9.120e-05, p_(FDR)_ = 0.0089), lateral temporal (β =−8.228e-05, p_(FDR)_ = 0.0289), superior lateral occipital (β = −1.781e-04, p_(FDR)_ = 0.0004) and middle frontal (β = −1.033e-04, p_(FDR)_ = 0.0109), see Supplementary Figure 12.

The observed associations remained significant after adjustment for the APOE ε4 status (Supplementary Table 9). Similarly, the voxel-wise analyses showed a negative correlation between higher levels of P-tau217 and hypoperfusion in the temporo-occipito-parietal areas (Figure 4(d)). As depicted in Supplementary Figure 11 D, further adjustment for the APOE ε4 status did not affect the observed voxel-wise associations.

### Correlations between GM-CBF and CSF biomarkers of axonal and synaptic integrity

**Figure 5.**
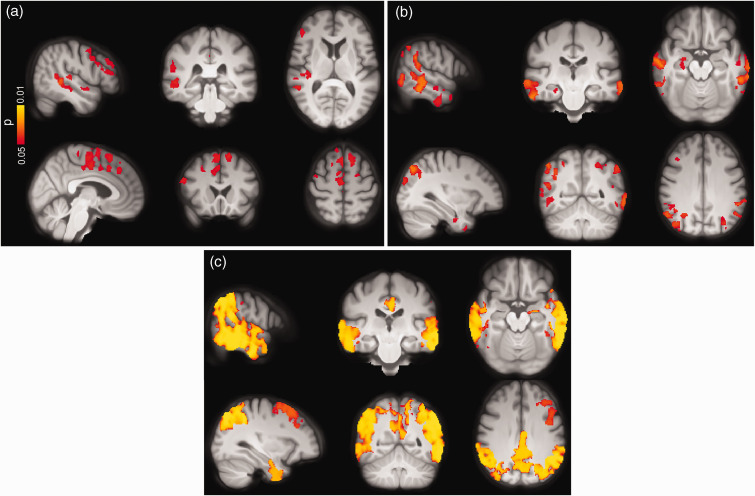
Voxel-wise associations between CBF and CSF levels of NfL and NPTX2/T-tau in CU individuals and those along the AD spectrum adjusted for age, sex and ASL sequence version. Increased levels of NfL were associated with lower CBF in both (a) CU subjects and (b) AD spectrum. (c) Decreasing levels of NPTX/T-tau were accompanied by CBF reduction mainly in temporo-occipito-parietal regions (p < 0.05, FWE corrected). Conventions as for Figure 3.

To study the associations between GM-CBF and markers of neurodegeneration, we used CSF levels of NfL, which increases in relation to ongoing axonal degeneration,^46^ and NPTX2/T-tau ratio which decreases with respect to synaptic degeneration.^47^ Using ROI-based analysis, no correlation was found between GM-CBF and NfL neither in CU nor in the individuals on the AD continuum (p_(FDR)_ > 0.05, see Supplementary Table 10) although there was a trend toward significance in middle frontal for the latter group (β = −0.0985, p_(FDR)_ = 0.0503). In contrast, the voxel-wise analysis revealed significant associations between higher CSF levels of NfL and lower GM-CBF in CU participants primarily affecting the right hemisphere in several frontal regions including frontal pole, inferior, middle and superior frontal gyri, precentral and paracingulate gyri, anterior cingulate and supplementary motor area as well as planum polar, superior/middle temporal and Heschl’s gyri (Figure 5(a)). In individuals along the AD continuum, widespread voxel-wise associations were observed between higher CSF concentrations of NfL and decreased perfusion mainly in temporo-occipito-parietal regions (Figure 5(b)). Further correction for the APOE ε4 status did not alter these associations (Supplementary Figure 13).

While CSF levels of NPTX2/T-tau were not correlated with GM-CBF in CU individuals (Supplementary Table 10), a positive association was observed between NPTX2/T-tau and GM-CBF in the AD continuum for lateral parietal (β = 0.0517, p_(FDR)_ = 0.0081), lateral temporal (β = 0.0553, p_(FDR)_ = 0.0081), superior lateral occipital (β = 0.0899, p_(FDR)_ = 0.0016) and middle frontal (β = 0.0815, p_(FDR)_ = 0.0003) ROIs, see Supplementary Figure 14. The observed correlations were largely corroborated by the voxel-wise analyses showing hypoperfusion with decreasing concentrations of NPTX2/T-tau (see Figure 5(c)). The ROI-based and voxel-wise associations remained significant after additional correction for the APOE ε4 genotype (Supplementary Figure 15).

Given the direct relationship between synaptic loss and cognitive impairment in AD,^48^ we also evaluated the associations between GM-CBF, and global cognition measured by MMSE.^49^ While GM-CBF alterations did not correlate with MMSE scores in CU individuals (see Supplementary Figure 16), hypoperfusion paralleled cognitive deficits in individuals on the AD continuum. For this analysis, the regression model was further controlled for the confounding effects of education. Cognitive impairment was significantly associated with hypoperfusion in the following cortical regions [lateral parietal (β = 4.113e-06, p_(FDR)_ = 0.0003), lateral temporal (β = 3.623e-06, p_(FDR)_ = 0.0031), superior lateral occipital (β = 6.849e-06, p_(FDR)_ = 0.00006) and middle frontal (β = 4.738e-06, p_(FDR)_ = 0.0003) ROIs]. Similarly, the voxel-wise analyses demonstrated positive correlations between decreased GM-CBF in multiple cortical regions and lower MMSE scores (Supplementary Figure 17). These associations remained intact following additional adjustment for the APOE ε4 genotype and cognitive status (see Supplementary Figure 18).

### Sensitivity analysis with coefficient of variation (COV) in ASL signal

To test whether the observed findings are affected by an inclusion bias due to the elimination of participants with macro-vascular artefacts (see Figure 1 and Supplementary Figure 2), we expanded the final study cohort by incorporating those subjects. Out of 48 individuals with high vascular artefacts, we included 46 participants, thereby increasing the sample size to N = 367. Two subjects were excluded due to missing tau-PET data. Vascular artefacts that are associated with prolonged arterial transit time (ATT) result in spatially inhomogeneous distribution of the ASL signal making the CBF measurement unreliable. The spatial coeffect of variation (sCoV) of ASL signal distribution in individuals with compromised cerebrovasculature provides a more robust measure than the CBF.^50^ Leveraging sCoV as the main outcome variable, we repeated the ROI-based analysis on between-group differences and its associations with selected markers of Aβ/tau pathology and synaptic degeneration. The results (Supplementary Table 11) were largely consistent with our findings using GM-CBF, indicating that exclusion of subjects with macro-vascular artefacts has minimal impact on the main findings.

### Staging of GM-CBF with respect to biomarkers of Aβ, tau and neurodegeneration

**Figure 6.**
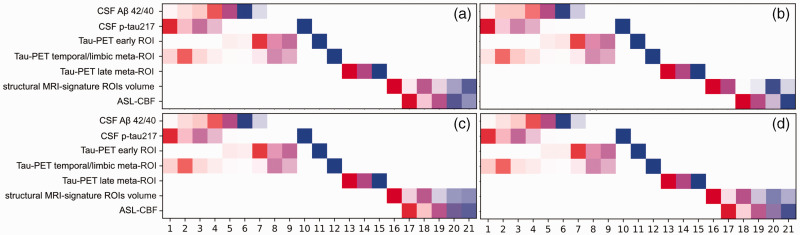
Progression pattern of AD predicted by the linear event progression model with four different estimations of the GM-CBF. In panel (a), the weighted mean GM-CBF was obtained in temporo-occipito-parietal regions demonstrating significant voxel-wise associations with tau-PET burden in the early ROI in the AD spectrum (see Figure 4(a)). The same mask of significant voxel-wise associations was used to extract CBF values in Aβ-negative controls. In panel (b), GM-CBF was derived as the mean of all predefined ROIs (see Figure 2). Panel (c) shows the temporal ordering of the weighted mean GM-CBF in precuneus and posterior cingulate along with other biomarkers and in panel (d) the total GM-CBF value was fed into the model. While the earliest disease stages comprised abnormalities in CSF markers of Aβ and tau, GM-CBF variations occurred during the latest stages of the disease progression. There is a small variation in the staging of the biomarkers between the four diagrams, particularly for the volumetric measure. This is due to the interdependence between variables. In panels a, c, and d the estimated GM-CBF values are highly collinear resulting in analogous positional variance diagrams while in panel b, the CBF values are slightly less collinear which in turn affects the staging of all the variables. At each stage, the accumulative probability for each biomarker going from one z-score to another is indexed by distinct colors i.e., red, magenta, and blue representing z-scores of 1, 2 and 3, respectively. Higher opacity indicates more confidence in the ordering.

A data-driven linear event progression modeling approach was applied to probabilistically assign individuals in Aβ-negative CU group and those along the AD continuum to one of 21 progressive stages (see Figure 6). The model predicted that abnormal CSF concentrations of Aβ42/40 and P-tau217 initiated the progression of the disease. This was followed by pathological deposition of tau-PET in early, temporal/limbic and late meta-ROIs, as well as neurodegeneration indicated by the atrophy in the AD signature regions. Only subsequent to these changes, did GM-CBF alterations become apparent. Importantly, regardless of how GM-CBF was estimated either from selected regions or globally (Figure 6(a) to (d)), the pattern of temporal ordering was consistent, suggesting a late onset for GM-CBF alterations.

## Discussion

The present study disentangles the multimodal associations of GM-CBF with different pathological markers in AD. We found that i) GM-CBF did not differ between the Aβ-negative and Aβ-positive CU groups. However in Aβ-positive CI patients, GM-CBF was markedly reduced compared to CU individuals independent of Aβ pathology; ii) the GM-CBF reduction were mainly localized in the temporo-occipito-parietal regions; iii) GM-CBF was not associated with markers of Aβ neither in CU nor in individuals on the AD continuum; iv) higher tau-PET uptake and increased concentrations of CSF P-tau217 were correlated with hypoperfusion patterns in the AD spectrum; v) abnormal levels of the NPTX2/T-tau ratio, a marker of synaptic degeneration, were associated with lower GM-CBF in the same group; vi) axonal degeneration as measured by higher CSF levels of NfL was correlated with lower perfusion in both CU and AD continuum; and vii) pathological reduction of GM-CBF was evident only after the abnormality of Aβ, tau and regional cortical atrophy. Taken together, these findings suggest that GM-CBF changes may not be an early event associated with aggregation of Aβ plaques during the preclinical phase of AD. Instead, these alterations are more tightly linked to tau pathology, synaptic and axonal degeneration.

Generally, the results of ROI-based analysis overlapped well with those of the voxel-wise analysis except for the association between NfL and GM-CBF that was only detected using the latter approach. This could be probably due to the more sensitive nature of the voxel-wise analysis that performs a voxel-by-voxel search as opposed to the ROI-based analysis wherein CBF values are averaged across many GM voxels. Consequently, mild GM-CBF alterations in parts of a *priori* defined ROIs may not reach the significance level. Additionally, in the CU group CBF-NfL correlation was observed in several regions which were not among the predefined ROIs and thereby could not have been detected by the ROI-based analysis.

The observed GM-CBF differences between Aβ-positive CI and the CU groups are consistent with the accumulating evidence demonstrating widespread hypoperfusion across different cortical regions in AD.^51–53^ Additional correction for the APOE ε4 genotype did not attenuate the observed effect sizes suggesting that between group GM-CBF differences are independent of APOE ε4 status. Conflicting results have been reported on CBF changes in CU individuals with elevated risk of AD. While some studies have shown patterns of hyperperfusion in CU APOE ε4 carriers,^54,55^ others have found no CBF changes in CU individuals with Aβ pathology.^13^ Our findings endorse the latter indicating that ASL-CBF is a marker of the disease severity, not directly related to Aβ deposition.

Lack of an association between CBF and Aβ burden, as suggested by our results, is in line with a previous study demonstrating no relationship between carotid and intracranial stenosis with global Aβ load in cognitively normal and cognitively impaired individuals with AD pathology.^56^ Similarly, it has been reported that long-lasting hypoperfusion due to unilateral occlusion of precerebral arteries does not promote aggregation of Aβ in non-demented humans.^57^ Another study, however, has found positive associations between hyperperfusion and increased Aβ-PET load in a small sample (N = 13) of Aβ-positive CU individuals.^15^ On the other hand, inverse associations between lower perfusion and increased Aβ-PET uptake have been reported across the spectrum of sporadic AD and in asymptomatic and mildly symptomatic subjects with autosomal dominant AD.^10,58^ While the observed discrepancy suggests that the potential role of tau should be taken into account when assessing the CBF-amyloid relationship, it may also arise from the differences in the sample size or acquisition of the ASL-CBF images. In the current study, we applied a pseudo-continuous sequence with background suppression and 3D GRASE readout, whereas in the latter two studies, a pulsed sequence was used, which is less sensitive and more prone to being influenced by the variations in ATT.^29^ Our findings on the negative CBF-tau associations mainly in the temporo-occipito-parietal cortex in the AD spectrum are in general agreement with previous studies comparing tau-PET uptake with glucose metabolism using FDG-PET,^59^ and neuropathological results showing higher tau pathology with increased concentrations of vascular endothelial growth factor, an indicator of hypoxia-induced hypoperfusion.^60^ A recent study with direct comparison of ASL-CBF and tau-PET has also shown CBF reduction in similar cortical regions.^18^ The underlying mechanisms linking tau pathology to CBF decline in AD are yet to be determined. Evidence from animal models has suggested that chronic lower perfusion promotes the accumulation of tau tangles.^61,62^ In contrast, it has been proposed that neurofibrillary tau tangles have detrimental effects on the integrity of the cortical microvasculature.^63^

In this study, the unique combination of imaging and CSF measures allowed us to explore the associations of ASL-CBF not only with Aβ and tau as the hallmarks of AD, but also with synaptic and axonal function. We found that lower CBF was associated with lower levels of the NPTX2/T-tau ratio, indicating that synaptic degeneration is accompanied by CBF reduction. NPTX2 is a marker of glutamatergic synaptic plasticity,^64^ which has been shown to be down-regulated in AD correlating with cognitive deterioration and hippocampal volume.^65^ Here, we used NPTX2/T-tau ratio as it has been shown to have a high classification accuracy in discriminating AD from normal controls and a strong correlation with cognition.^47^

Elevated levels of NfL were correlated with decreased GM-CBF in both CU participants and in the AD continuum. Although there are no published reports on such associations, a few studies have found a link between higher CSF or plasma levels of NfL with cortical hypometabolism in AD.^66,67^ Further, another study has reported longitudinal associations between higher concentrations of NfL and decline in several neuroimaging measures including FDG-PET.^68^ Collectively, our findings suggest that CBF alterations in the AD spectrum are elicited by the spread of tau tangles, synaptic dysfunction and axonal degeneration.

We found that GM-CBF reduction in widespread cortical regions was associated with worse cognitive performance in the AD continuum. Several studies have investigated the relationship between ASL-CBF and cognition reporting similar results.^69,70^ Synaptic failure is thought to be one of the determinants of cognitive impairment in AD.^48^ Our findings support this hypothesis and augments previous results by displaying simultaneous associations of hypoperfusion with synaptic loss and cognitive deterioration. These findings also seem in line with previous longitudinal reports showing that hypoperfusion particularly in posterior regions is related to the rate of future cognitive decline both in AD patients and healthy elderly ^71,72^ although in the latter study the authors also found reduced baseline CBF in posterior cingulate when comparing healthy controls with stable vs deteriorated cognition.^72^ This contradictory result could be due to lack of stratification of their controls based on Aβ pathology. Altogether, our findings suggest that CBF may be less sensitive in early detection of brain changes but may prove valuable in reflecting the disease severity.

Whether perfusion disruption is a cause or consequence of AD remains unclear.^73,74^ In a pioneering study, Iturria-Medina et al., reconstructed AD-abnormality trajectories and suggested an early role of vascular dysregulation in the AD development, which was followed by changes in Aβ deposition, metabolic dysfunction, functional impairment and structural atrophy.^75^ In contrast, we found that anomalous concentrations of Aβ and tau along with cortical volume loss preceded GM-CBF deficiency in AD. The observed disparity between our findings and those of Iturria-Medina’s study on the contribution of CBF disturbances in the pathological cascade of AD may arise from several different factors. First, in the ADNI dataset employed in that study, the ASL scans were acquired using a PICORE-PASL sequence, increasing the probability of incomplete delivery of the labeled blood to the brain.^29^ Patients with compromised vasculature suffer from this effect more than individuals with relatively healthy vasculature.^50^ Consequently, the results of their study might be more sensitive to the macrovascular changes while our results are more influenced by changes in localized tissue perfusion.^76^ From this perspective, cerebrovascular hemodynamics could probably be an early biomarker — for any type of dementia including AD — whereas localized tissue perfusion is a rather late biomarker for AD.

Secondly, many of the early- and late-onset MCI patients in their study were Aβ-negative individuals. On the contrary, we excluded all patients without amyloid pathology (see Figure 1) and focused on Aβ-positive participants on the AD continuum together with Aβ-negative controls. The rationale behind this decision was to investigate the relationship between GM-CBF and the neuropathological mechanisms in AD while the underlying pathology in Aβ-negative MCI patients is independent of amyloid and tau accumulation. Moreover, the disease progression modeling applied to this study assumes that patients will dynamically evolve through the stages of the same disease. Inclusion of Aβ-negative MCI patients would violate this assumption since these patients will likely convert to non-AD dementia.

It may be questioned how concomitant cerebral amyloid angiopathy (CAA) affects the observed findings. While CAA is associated with decreased concentrations of both CSF Aβ42 and Aβ40, only CSF levels of Aβ42 is reduced in AD.^77^ Therefore, compared to CAA, the CSF levels of Aβ42/Aβ40 is decreased in AD. This facilitates differentiating between the two diseases and confirms absence of CAA pathology in our cohort. The cross-sectional nature of this study did not allow us to determine the temporal relationship of ASL-CBF with each of the imaging and CSF measures. Further research is thus warranted to elucidate the longitudinal associations between CBF and markers of amyloid, tau, synaptic and axonal integrity. Further in the present study, we only employed PV-corrected GM-CBF estimates. As such, the obtained results may not apply to CBF fluctuations in WM. An acquisition protocol with multiple PLDs is preferred for reliable quantification of WM-CBF which inevitably results in a longer acquisition time making it suboptimal in clinical populations. Although the increase in time can be lessened using simultaneous multi-slice imaging,^78^ this was not implemented in our ASL sequence. Here, robust estimation of WM-CBF was not feasible due to our single-PLD acquisition and the target study population of elderly patients. Consequently, we opted to proceed with GM-CBF estimates. Another potential limitation of the preset study is the relatively short labeling duration (1500 ms) of the ASL sequence which may increase the ATT. We discarded 48 ASL scans from a sample of 539 Aβ-negative controls and Aβ-positive individuals on the AD spectrum due to macro-vascular artefacts and prolonged ATT (see Figure 1 and Supplementary Figure 2 B). Although this may have caused a selection-bias in our final sample, it’s impact was minimal affecting less than 9% of the ASL scans. Furthermore, sensitivity analysis using sCoV confirmed that the observed findings are largely independent of vascular burden (Supplementary Table 11).

Importantly, the between-group differences in GM-CBF and its associations with tau, synaptic and axonal dysfunction were primarily localized in temporo-occipito-parietal regions. Coincidentally, parts of these regions overlap with cerebral watershed areas that are boundary-zones between major cerebral arteries. Given the vulnerability of these border zones to hypoperfusion, caution should be exerted when interpreting the relationships between reduced GM-CBF and AD pathology.

In conclusion, these results provide in vivo evidence indicating that tau aggregates and neurodegeneration, but not Aβ, are closely connected to ASL-CBF alterations across the AD continuum. This suggests that cerebral hypoperfusion, at least measured by ASL, does not stimulate the development of AD pathology but rather is a late reactive change. However, it should be stressed that due to the limited spatial resolution of ASL, the observed findings may not represent the dynamics of blood flow within the cerebral capillaries. The discrepancy between these results and some of the previous studies^72,75^ that suggest ASL-CBF changes occur at early stages of AD, highlights the complexity of this disease. Future CBF studies should investigate the temporal ordering of biomarker abnormalities in conjunction with their pathological interactions to enhance our knowledge of AD progression.

## Supplemental Material

sj-pdf-1-jcb-10.1177_0271678X221141139 - Supplemental material for Gray matter hypoperfusion is a late pathological event in the course of Alzheimer’s disease Click here for additional data file. Supplemental material, sj-pdf-1-jcb-10.1177_0271678X221141139 for Gray matter hypoperfusion is a late pathological event in the course of Alzheimer’s disease by Khazar Ahmadi, Joana B Pereira, David Berron, Jacob Vogel, Silvia Ingala, Olof T Strandberg, Shorena Janelidze, Frederik Barkhof, Josef Pfeuffer, Linda Knutsson, Danielle van Westen, Sebastian Palmqvist, Henk JMM Mutsaerts and Oskar Hansson in Journal of Cerebral Blood Flow & Metabolism
