# Supplementary material for: Gray matter hypoperfusion is a late pathological event in the course of Alzheimer’s disease
Source: J Cereb Blood Flow Metab. 2022 Nov 22;43(4):565–80. doi: 10.1177/0271678X221141139 (PMC10063832; doi:10.1177/0271678X221141139)
Supplement: sj-pdf-1-jcb-10.1177_0271678X221141139 - Supplemental material for Gray matter hypoperfusion is a late pathological event in the course of Alzheimer’s disease [file sj-pdf-1-jcb-10.1177_0271678X221141139.pdf]

Supplemental material

	Aβ-negative CU	Aβ-positive CU	Aβ-positive CI	Non-AD	Total included N
Present study	142	60	119	60 + 132 = 192	321
Previous paper by Palmqvist et al., (2020)	224	77	213	86 + 99 = 185	699
Overlapping participants	102	42	103	59 + 69 = 128	

Supplementary Table 1. Number of participants per group in the current study and our earlier publication (Palmqvist et al., 2020) in conjunction with quantification of the overlapping participants. In our study, Aβ-negative MCI patients (N = 60) were merged within the non-AD group (see the caption of Figure 1), whereas they were considered as a separate entity in the aforementioned paper (N = 86). Here, the non-AD group is displayed as the sum of number of Aβ-negative MCI patients and those with diagnoses other than AD. Note that the non-AD group was excluded from the main analyses in our study. Overall, PET and structural MRI scans, as well as CSF biomarkers from 247 participants (102 + 42 + 103 = 247) were independently utilized in these two studies. However, no ASL data was included in that publication. The difference in the number of participants per group between the two studies is mostly due to several exclusion criteria in our study (see Figure 1) resulting in a smaller sample size per group. On the other hand, the difference between the number of overlapping participants and the number of included participants per group in the present study (1st and 3rd rows) indicates recruitment of new participants as the BioFINDER-2 study is still ongoing.

	**Amyloid-negative CU**			**Amyloid-positive CU**			**Amyloid-positive CI**		
	**Pre N = 64**	**Post N = 78**	**p value**	**Pre N = 30**	**Post N = 30**	**p value**	**Pre N = 56**	**Post N = 63**	**p value**
**Male/female**	33/31	31/47	0.1687	9/21	17/13	0.149	25/31	24/39	0.1872
**Age**	63.90 (10.18)	66.82 (10.55)	0.0964	71.51 (8.90)	71.86 (7.95)	0.872	72.61 (6.63)	73.17 (7.11)	0.658
**Education**	12.53 (3.22)	12.62 (4.03)	0.888	13.6 (4.40)	12.24 (3.57)	0.198	12.9 (4.67)	12.54 (4.25)	0.669
**APOE ε4 carriers %**	32.81%	32.05%	0.555	80%	63.33%	0.445	69.6%	77.7%	0.2864
**MMSE**	28.90 (1.16)	28.90 (1.21)	0.988	28.60 (1.47)	28.63 (1.47)	0.930	23.58 (4.45)	23.71 (4.38)	0.877

Supplementary Table 2. Demographic characteristics of the participants who were scanned with either pre- or post-updated versions of the ASL sequence. Data are presented

as mean values followed by (standard deviations). In each group, the demographic factors were compared between sequence versions using X^2 or independent t-tests.

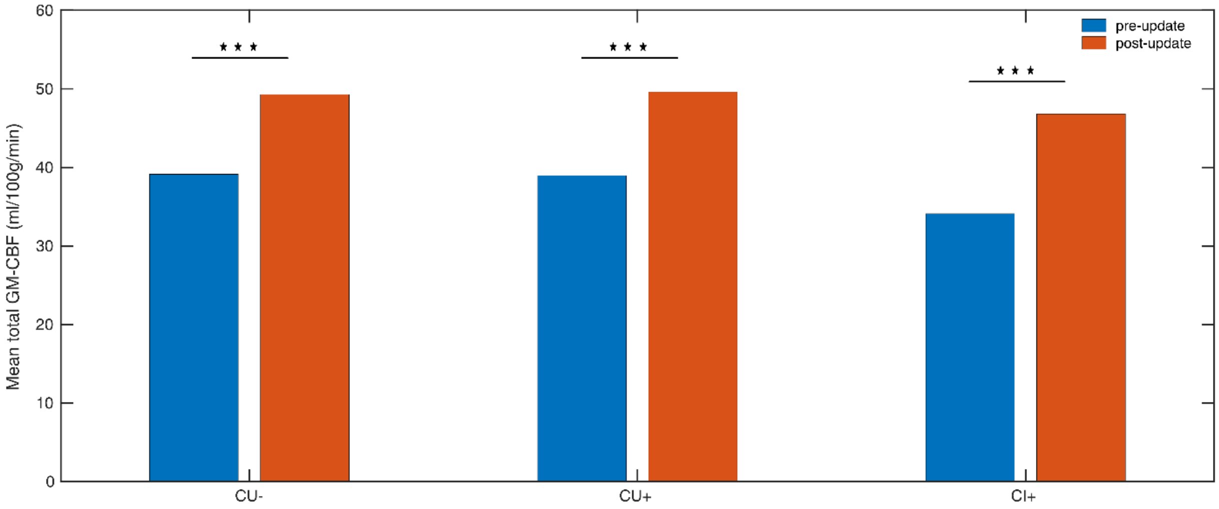

Supplementary Figure 1. Comparison of the total GM-CBF between pre- and post-updated versions of the ASL sequence in Aβ-negative and Aβ-positive cognitively unimpaired (CU- and CU+) and Aβ-positive cognitively impaired (CI+) groups. Those who were scanned following the sequence update show higher global GM-CBF.

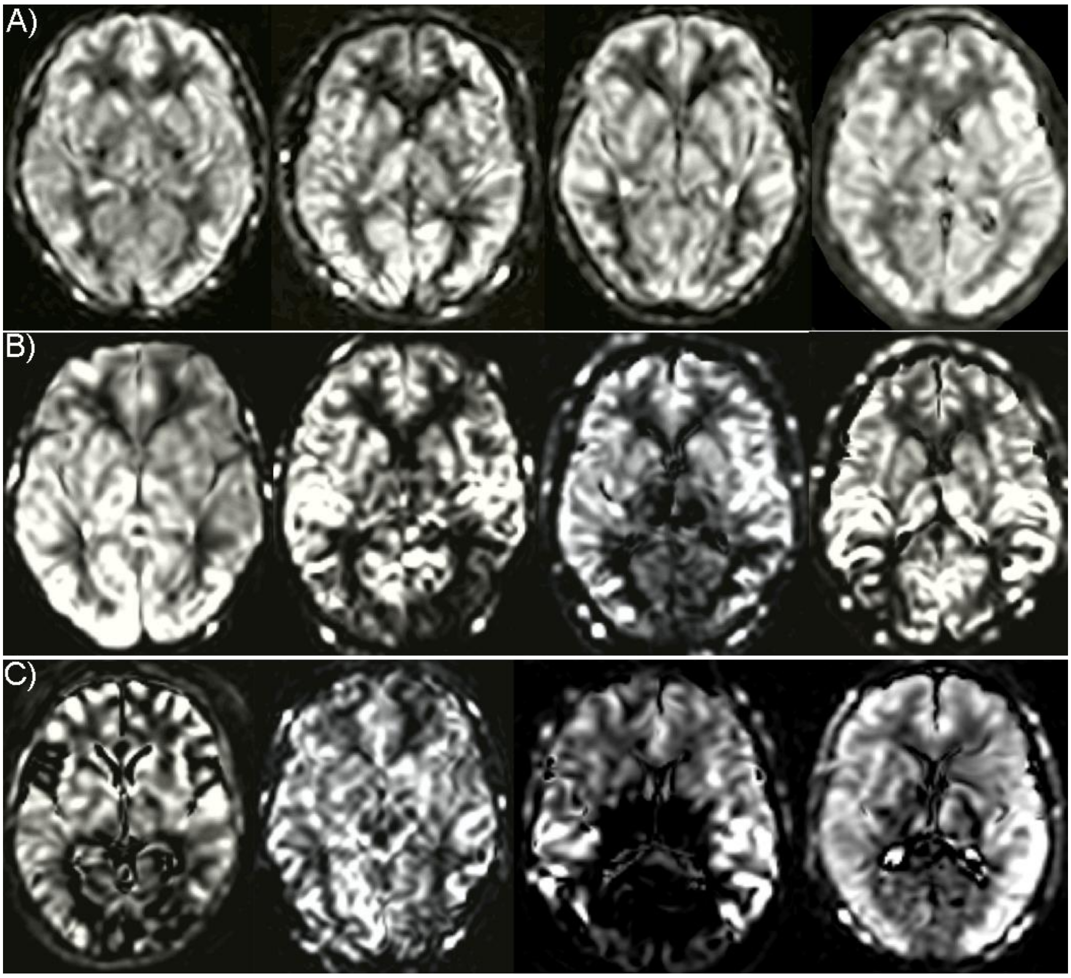

Supplementary Figure 2. Examples of ASL scans with A) CBF contrast, B) vascular burden and C) other types of artefacts. Only ASL images with CBF contrast were used for the main analysis. Although some vascular component was removed from the data by discarding images with macro-vascular artefacts, this exclusion effect was minimal since less than 9% of the initial cohort (48 out of 539 Aβ-negative and Aβ-positive individuals) were labeled as having vascular artefacts.

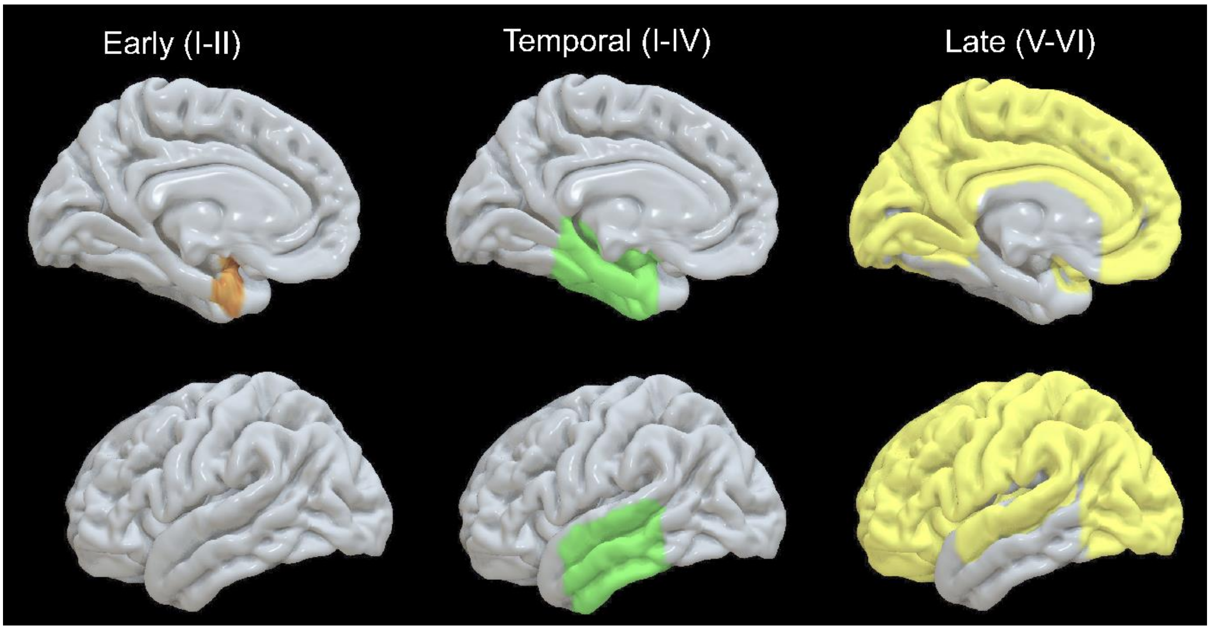

Supplementary Figure 3. Tau-PET ROIs corresponding to Braak stages I-II, I-IV and V-VI (left, middle and right panels, respectively). Top and bottom panels display the lateral and medial views. For the sake of visualization, the bilateral ROIs are shown on the left hemisphere. The early ROI (I-II) consisted of entorhinal cortex while the remaining meta-ROIs encompassed the following sub-ROIs: I-IV (amygdala, fusiform gyrus, inferior/middle temporal gyri, parahippocampus, and entorhinal cortex); V-VI (anterior/posterior cingulate, inferior/superior frontal, inferior/superior parietal, insular, lateral/medial occipital, lingual gyrus, middle frontal, orbitofrontal cortex, paracentral cortex, precentral/postcentral gyri, precuneus, superior temporal gyrus, and supramarginal gyrus).

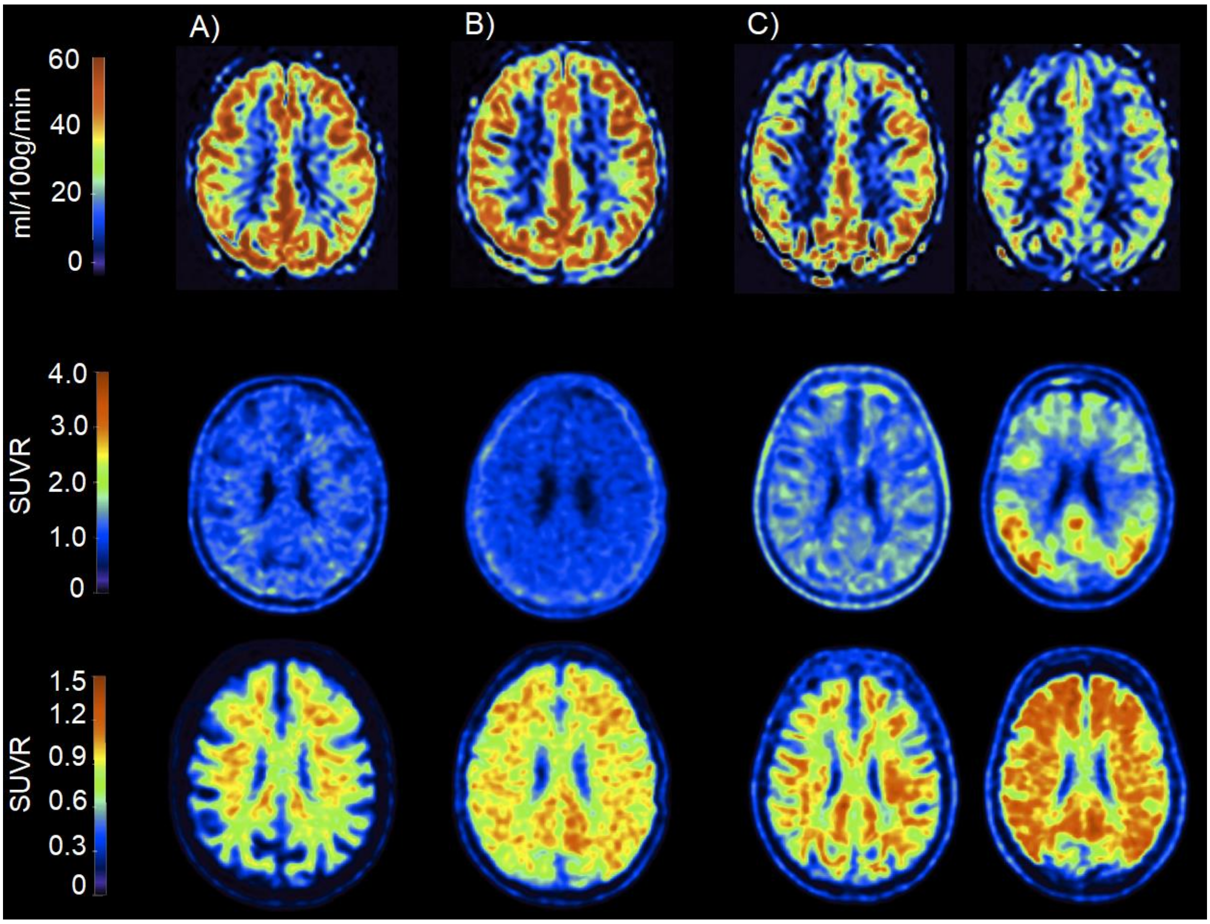

Supplementary Figure 4. Color-coded maps of ASL-CBF, tau- and Aβ-PET scans (top, middle and bottom panels, respectively) in representative A) Aβ-negative CU, B) Aβ-positive CU and C) Aβ-positive CI participants. Note that panel C represents 2 images per modality. The images on the left side of this panel belong to a participant with MCI while those on the far-right side shows a subject with AD-dementia.

	Aβ-negative CU	Aβ-positive CU	Aβ-positive CI
**Total N**	142	60	119
**Global ASL-CBF**	142<br>(44.70 ± 11.47)	60<br>(44.27 ± 12.28)	119<br>(40.82 ± 10.83)
**Tau-PET uptake in late meta-ROI**	142<br>(1.039 ± 0.09)	60<br>(1.080 ± 0.135)	119<br>(1.32 ± 0.35)
**P-tau217**	112<br>(51.10 ± 28.78)	48<br>(224.45 ± 139.40)	99<br>(467.46 ± 282.41)
**Global Aβ-PET uptake**	132<br>(0.46 ± 0.034)	57<br>(0.68 ± 0.14)	56<br>(0.76 ± 0.16)
**Aβ42/40 ratio**	123<br>(1.064 ± 0.16)	56<br>(0.53 ± 0.11)	116<br>(0.48 ± 0.12)
**NPTX2/T-tau ratio**	116<br>(2.03 ± 0.67)	54<br>(1.26 ± 0.44)	113<br>(0.68 ± 0.31)
**Log-transformed NfL**	121<br>(2.92 ± 0.21)	55<br>(2.99 ± 0.22)	115<br>(3.19 ± 0.26)

Supplementary Table 3. Available data per group and biomarker with corresponding (mean ± standard deviations) values. Although the Aβ42/40 ratio values obtained by Lumipulse assay were used for stratification of the participants, they were excluded from the subsequent analysis on the association of CBF and CSF Aβ42/40. As such, the number of available data for this marker is less than the total number of the study participants.

	Coefficient	FDR-corrected p value
**Lateral Parietal**	-0.0025	0.8535
**Lateral temporal**	-0.0142	0.6290
**Superior lateral occipital**	0.0198	0.6290
**Middle frontal**	0.0041	0.8535
**Medial parietal**	-0.0153	0.6290
**Medial temporal**	-0.0162	0.6290

Supplementary Table 4. Group differences in GM-CBF between Aβ-negative and Aβ-positive CU participants, corrected for the confounding effects of age, sex and ASL sequence version. No significant between-group GM-CBF differences are observed.

	Aβ-negative CU vs. Aβ-positive CU	Aβ- negative CU vs. Aβ-positive CI	Aβ-positive CU vs. Aβ-positive CI
**Lateral Parietal**	β = -0.0086<br>$p_{(FDR)}$ = 0.6753	β = -0.0386<br>$p_{(FDR)}$ = 0.0164*	β = -0.0327<br>$p_{(FDR)}$ = 0.1054
**Lateral temporal**	β = -0.0220<br>$p_{(FDR)}$ = 0.5021	β = -0.0538<br>$p_{(FDR)}$ = 0.0052**	β = -0.0300<br>$p_{(FDR)}$ = 0.1375
**Superior lateral occipital**	β = 0.0199<br>$p_{(FDR)}$ = 0.5108	β = -0.0600<br>$p_{(FDR)}$ = 0.0055**	β = -0.0814<br>$p_{(FDR)}$ = 0.0030**
**Middle frontal**	β = 0.0065<br>$p_{(FDR)}$ = 0.7102	β = -0.0519<br>$p_{(FDR)}$ = 0.0055**	β = -0.0611<br>$p_{(FDR)}$ = 0.0035**
**Medial parietal**	β = -0.0203<br>$p_{(FDR)}$ = 0.5108	β = -0.0114<br>$p_{(FDR)}$ = 0.6204	β = 0.0028<br>$p_{(FDR)}$ = 0.8817
**Medial temporal**	β = -0.0223<br>$p_{(FDR)}$ = 0.5021	β = -0.0036<br>$P_{(FDR)}$ = 0.7723	β = 0.0138<br>$p_{(FDR)}$ = 0.3847

Supplementary Table 5. Between group differences in GM-CBF, adjusted for age, sex, ASL sequence version, and the APOE ε4 status. While there is no GM-CBF differences between Aβ-negative and Aβ-positive CU controls, lower GM-CBF is observed in superior lateral occipital and middle frontal gyrus in Aβ-positive CI patients compared to Aβ-positive CU individuals. In addition to the aforementioned ROIs, GM-CBF is also decreased in lateral parietal and lateral temporal regions when comparing Aβ-negative CU vs Aβ-positive CI participants.

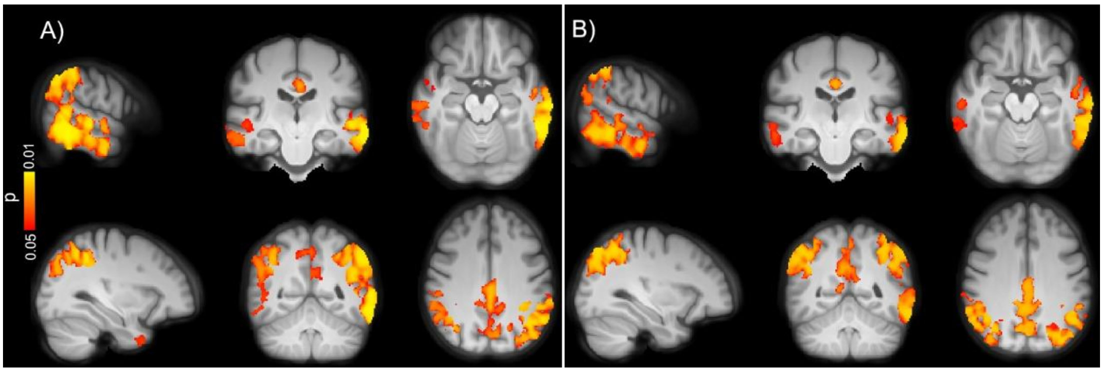

Supplementary Figure 5. Voxel-wise group differences in GM-CBF between A) Aβ-negative CU vs. Aβ-positive CI and B) Aβ-positive CU vs. Aβ-positive CI individuals corrected for age, sex, ASL sequence version and the APOE ε4 status. In line with the main findings, the pattern of hypoperfusion covers the temporo-occipito-parietal regions ($p < 0.05$, FWE-corrected). Conventions as for Figure 3.

	Association of GM-CBF with CSF Aβ42/40		Association of GM-CBF with global Aβ-PET uptake	
	CU	AD spectrum	CU	AD spectrum
**Lateral Parietal**	β = 0.0023<br>$p_{(FDR)}$ = 0.9972	β = 0.0142<br>$p_{(FDR)}$ = 0.9216	β = -0.0038<br>$p_{(FDR)}$ = 0.9607	β = -0.0788<br>$p_{(FDR)}$ = 0.3797
**Lateral temporal**	β = 0.0223<br>$p_{(FDR)}$ = 0.7752	β = -0.0657<br>$p_{(FDR)}$ = 0.7613	β = -0.0408<br>$p_{(FDR)}$ = 0.9607	β = -0.1124<br>$p_{(FDR)}$ = 0.3797
**Superior lateral occipital**	β = -0.0370<br>$p_{(FDR)}$ = 0.7752	β = -0.0822<br>$p_{(FDR)}$ = 0.7613	β = 0.1198<br>$p_{(FDR)}$ = 0.5045	β = -0.0004<br>$p_{(FDR)}$ = 0.9957
**Middle frontal**	β = -0.0130<br>$p_{(FDR)}$ = 0.9521	β = -0.0073<br>$p_{(FDR)}$ = 0.9216	β = 0.0163<br>$p_{(FDR)}$ = 0.9607	β = -0.0960<br>$p_{(FDR)}$ = 0.3797
**Medial parietal**	β = -0.0302<br>$p_{(FDR)}$ = 0.7752	β = -0.0717<br>$p_{(FDR)}$ = 0.7613	β = 0.0034<br>$p_{(FDR)}$ = 0.9607	β = -0.0317<br>$p_{(FDR)}$ = 0.8485
**Medial temporal**	β = 0.00008<br>$p_{(FDR)}$ = 0.9972	β = -0.0261<br>$p_{(FDR)}$ = 0.9216	β = -0.0167<br>$p_{(FDR)}$ = 0.9607	β = -0.0208<br>$p_{(FDR)}$ = 0.8485

Supplementary Table 6. Association of GM-CBF with Aβ pathology in CU individuals and those on the AD spectrum adjusted for age, sex and ASL sequence version. GM-CBF is not associated neither with CSF levels of Aβ42/40 nor with global Aβ-PET load in none of the studied groups.

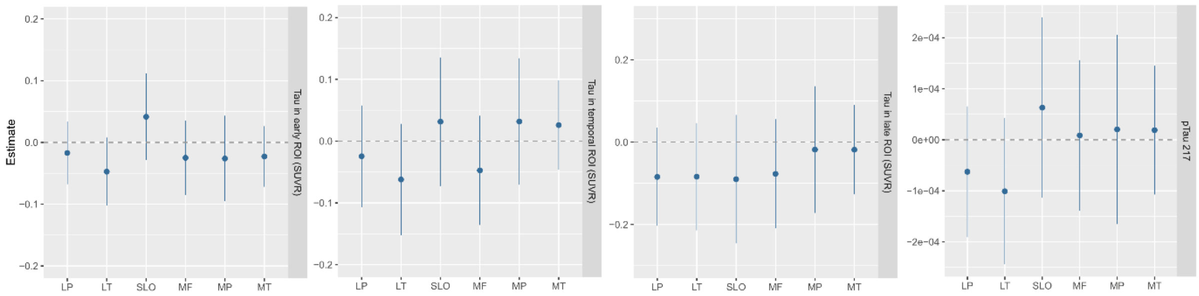

Supplementary Figure 6. Association of GM-CBF with tau pathology, corrected for age, sex and ASL sequence version in CU individuals. No association is observed between CSF levels of P-tau 217 and tau-PET uptake in any of the early, temporal and late ROIs with GM-CBF in this group ($p > 0.05$ uncorrected). LP = lateral parietal, LT = lateral temporal, SLO = superior lateral occipital, MF = middle frontal, MP = medial parietal and MT = medial temporal.

	Normalized GM-CBF in			
	**Lateral parietal**	**Lateral temporal**	**Superior lateral occipital**	**Middle frontal**
**Early ROI (SUVR)**	β = -0.0432 $p_{(FDR)}$ = 0.0299*	β = -0.0528 $p_{(FDR)}$ = 0.0172*	β = -0.0888 $p_{(FDR)}$ = 0.0017**	β = -0.0423 $p_{(FDR)}$ = 0.044*
**Temporal meta-ROI (SUVR)**	β = -0.0545 $p_{(FDR)}$ = 0.0001***	β = -0.0632 $p_{(FDR)}$ = 0.00005***	β = -0.0917 $p_{(FDR)}$ = 0.00005***	β = -0.0427 $p_{(FDR)}$ = 0.0105*
**Late meta-ROI (SUVR)**	β = -0.1169 $p_{(FDR)}$ = 0.00006***	β = -0.0887 $p_{(FDR)}$ = 0.00208**	β = -0.1341 $p_{(FDR)}$ = 0.00079***	β = -0.0995 $p_{(FDR)}$ = 0.00149**

Supplementary Table 7. Associations of GM-CBF in four pre-defined ROIs with tau uptake in early, temporal, and late ROIs in the AD continuum, following additional adjustment for CSF levels of Aβ42/40. The associations remain intact after this correction suggesting that the relationship between GM-CBF and tau-PET load is independent of amyloid pathology.

**(SUVR)**	Normalized GM-CBF in			
	**Lateral parietal**	**Lateral temporal**	**Superior lateral occipital**	**Middle frontal**
**Early ROI**	β = -0.0402 p = 0.0431*	β = -0.0488 p = 0.0237*	β = -0.0579 p = 0.0293*	
**Temporal meta-ROI**	β = -0.0552 $p_{(FDR)}$ = 0.0009**	β = -0.0648 $p_{(FDR)}$ = 0.0004***	β = -0.0732 $p_{(FDR)}$ = 0.0009**	
**Late meta-ROI**	β = -0.1168 $p_{(FDR)}$ = 0.0002***	β = -0.0886 $p_{(FDR)}$ = 0.0197*	β = -0.0916 $p_{(FDR)}$ = 0.0239*	β = -0.0759 $p_{(FDR)}$ = 0.0239*

Supplementary Table 8. Relationship between GM-CBF in the pre-defined ROIs and tau SUVR in early, temporal, and late ROIs in the AD continuum, adjusted for age, sex, ASL sequence version, and cognitive status. Note that the observed associations between GM-CBF and tau burden in the early ROI remained statistically significant after adjustment for the cognitive status but did not survive FDR correction.

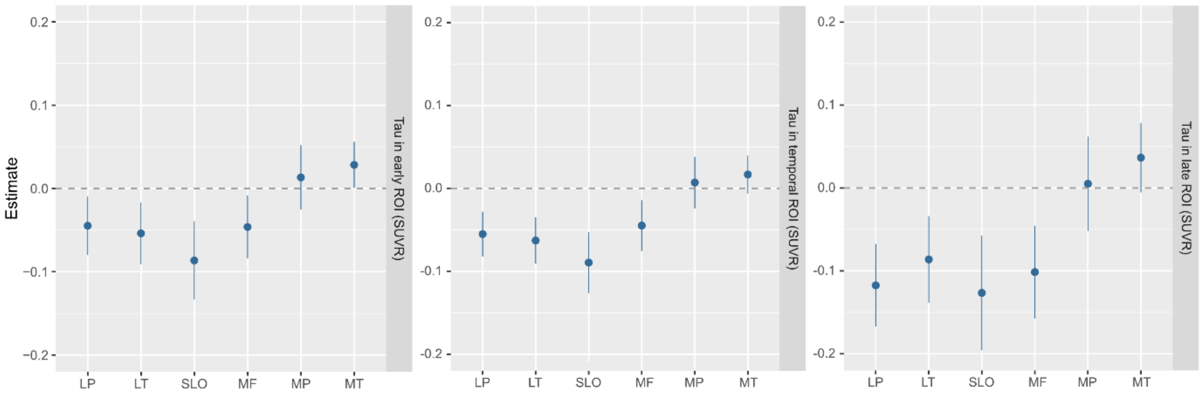

Supplementary Figure 7. Regression coefficients (β) for the associations between GM-CBF in a priori ROIs and the three tau-PET ROIs in the AD continuum, adjusted for age, sex, ASL sequence version, and APOE ε4 status. Except for medial parietal and medial temporal ROIs, all estimated coefficients are significant at $p_{(FDR)} \leq 0.05$. Conventions as for Supplementary Figure 6.

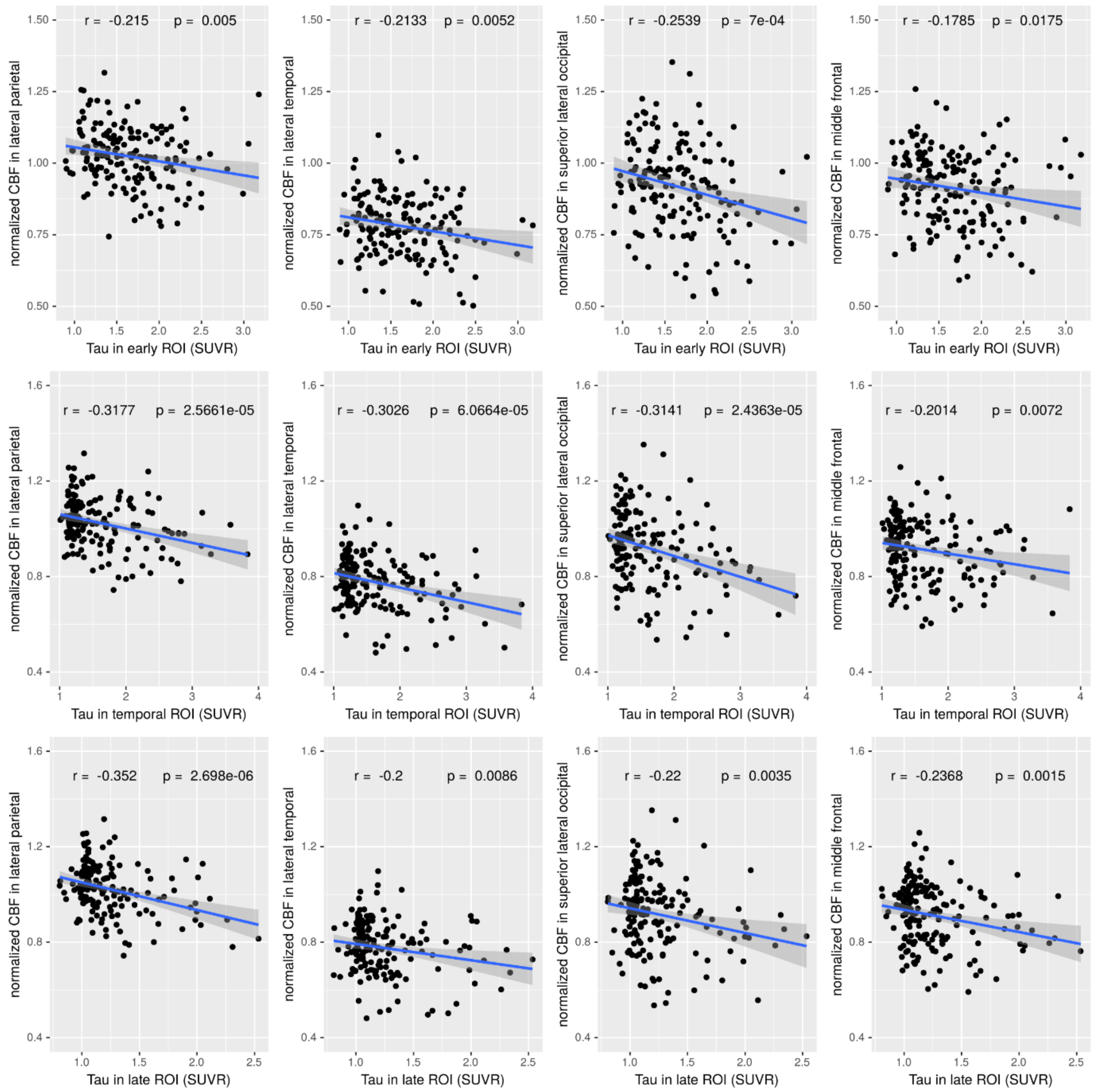

Supplementary Figure 8. Overall significant associations between GM-CBF in the pre-defined regions and tau load in early (top panel), temporal (middle panel) and late (bottom panel) ROIs in the AD continuum. The CBF in each pre-defined ROI is normalized to the CBF of the precentral gyrus (see Methods). The translucent area around the regression line indicates the 95% confidence interval for the regression coefficient. Note that the annotated statistical parameters (r and the corresponding uncorrected p values) are based on the correlation of CBF and tau-PET load without adjusting for confounding variables.

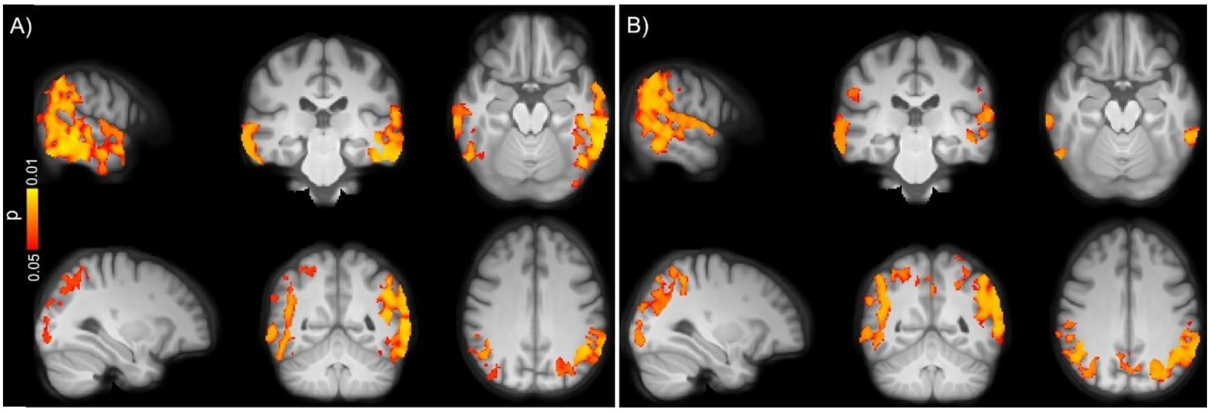

Supplementary Figure 9. Voxel-wise associations between GM-CBF and tau load in A) temporal and B) late meta-ROIs in the AD spectrum, corrected for age, sex, ASL sequence version and cognitive status. Clusters with markedly decreased GM-CBF are visible in temporo-occipito-parietal ROIs ($p < 0.05$, FWE corrected). Conventions as for Figure 3.

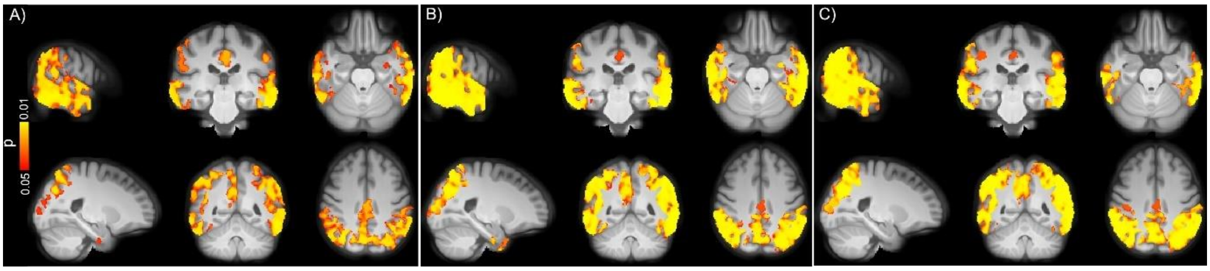

Supplementary Figure 10. Voxel-wise associations between GM-CBF and tau uptake in A) early, B) temporal and C) late meta-ROIs in the AD continuum following further correction for CSF levels of Aβ42/40. The observed widespread associations indicate that the relationship between CBF in temporo-occipito-parietal cortex and tau pathology is independent of amyloid deposition ($p < 0.05$, FWE corrected). Conventions as for Figure 3.

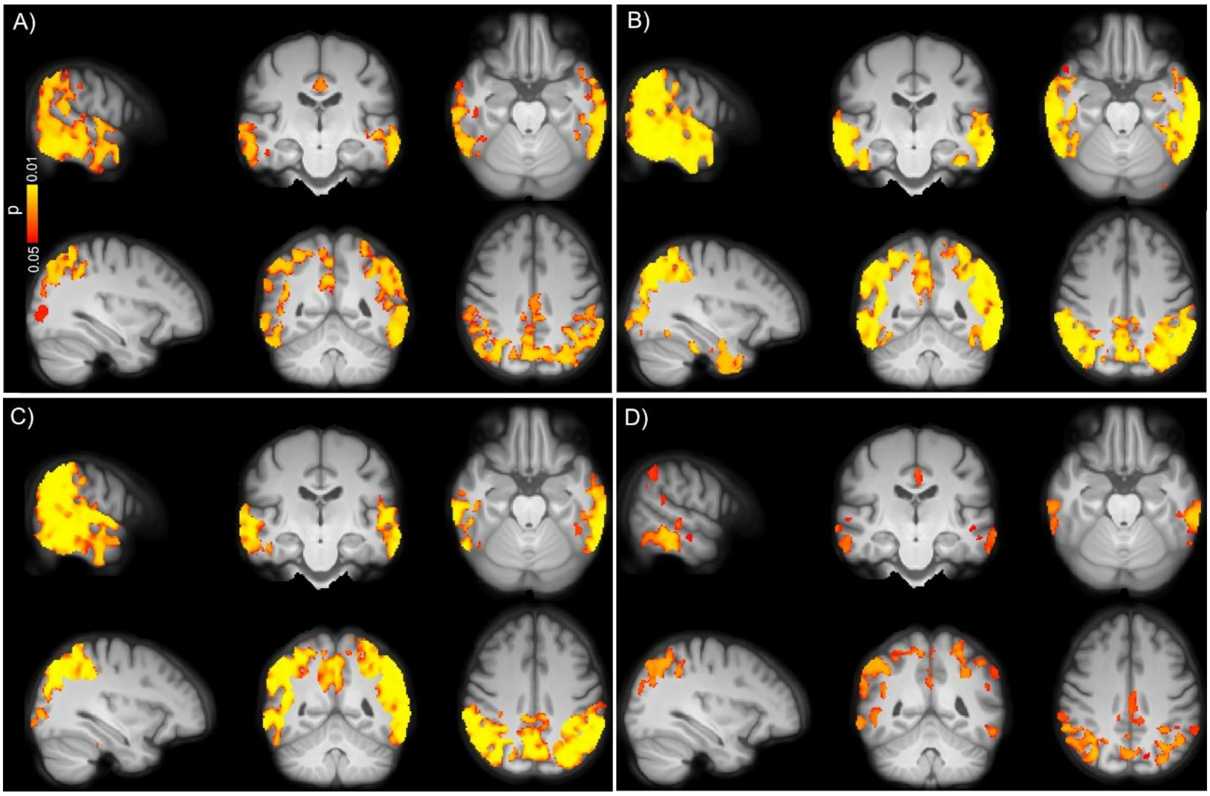

Supplementary Figure 11. Voxel-wise associations between GM-CBF and tau load in A) early B) temporal, C) late ROIs and D) CSF concentrations of P-tau 217 in the AD spectrum, adjusted for age, sex, ASL sequence version and APOE ε4 status. Additional adjustment for the APOE ε4 status does not affect extensive hypoperfusion in temporo-occipito-parietal regions ($p < 0.05$, FWE corrected). Conventions as for Figure 3.

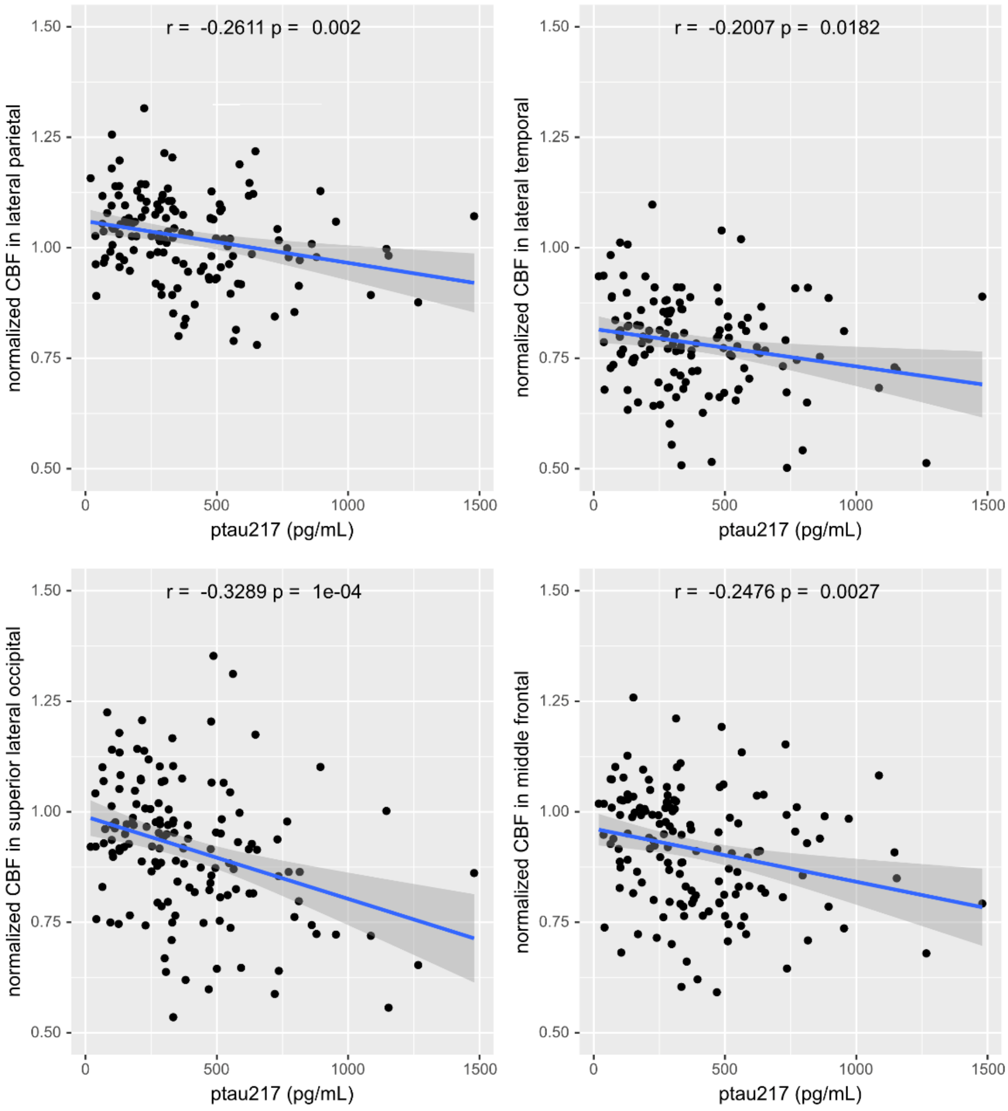

Supplementary Figure 12. ROI-based correlations between GM-CBF and P-tau217. Lower CBF is correlated with higher levels of P-tau217 in the AD spectrum. Conventions as for Supplementary Figure 8.

	**Coefficient**	**FDR-corrected p value**
**Lateral Parietal**	-8.966e-05	0.0102*
**Lateral temporal**	-8.250e-05	0.0290*
**Superior lateral occipital**	-1.787e-04	0.0004***
**Middle frontal**	-1.032e-04	0.0113*
**Medial parietal**	5.595e-06	0.8820
**Medial temporal**	3.175e-05	0.3127

Supplementary Table 9. Association of GM-CBF with P-tau217 following further correction for APOE ε4 status. In line with the main findings, significant associations are

found in lateral parietal/temporal, superior lateral occipital and middle frontal cortices indicating that CBF-P-tau 217 relationship is independent of APOE ε4 status.

	**Association of GM-CBF with log-transformed NfL**		**Association of GM-CBF with NPTX2/T-tau**
	**CU**	**AD spectrum**	**CU**
**Lateral Parietal**	β = 0.0501<br>$p_{(FDR)}$ = 0.2464	β = -0.0585<br>$p_{(FDR)}$ = 0.2497	β = 0.0063<br>$p_{(FDR)}$ = 0.7124
**Lateral temporal**	β = 0.0516<br>$p_{(FDR)}$ = 0.2464	β = -0.0505<br>$p_{(FDR)}$ = 0.2991	β = -0.0110<br>$p_{(FDR)}$ = 0.7124
**Superior lateral occipital**	β = 0.0778<br>$p_{(FDR)}$ = 0.2464	β = -0.0224<br>$p_{(FDR)}$ = 0.7518	β = 0.0117<br>$p_{(FDR)}$ = 0.7124
**Middle frontal**	β = 0.0253<br>$p_{(FDR)}$ = 0.6383	β = -0.0985<br>$p_{(FDR)}$ = 0.0503	β = 0.0217<br>$p_{(FDR)}$ = 0.7078
**Medial parietal**	β = 0.0736<br>$p_{(FDR)}$ = 0.2464	β = -0.0196<br>$p_{(FDR)}$ = 0.7518	β = -0.0134<br>$p_{(FDR)}$ = 0.7124
**Medial temporal**	β = 0.0050<br>$p_{(FDR)}$ = 0.8802	β = 0.0040<br>$p_{(FDR)}$ = 0.8861	β = -0.0020<br>$p_{(FDR)}$ = 0.8634

Supplementary Table 10. Associations of GM-CBF with CSF markers of axonal and synaptic integrity in CU individuals and subjects on the AD continuum, corrected for ag, sex and ASL sequence version. GM-CBF is not correlated with neither NfL nor NPTX2/T-tau in the CU individuals whereas in participants along the AD spectrum, the associations are insignificant only for NfL ($p > 0.05$ FDR-corrected). However, a trend towards significance is observed in the middle frontal cortex.

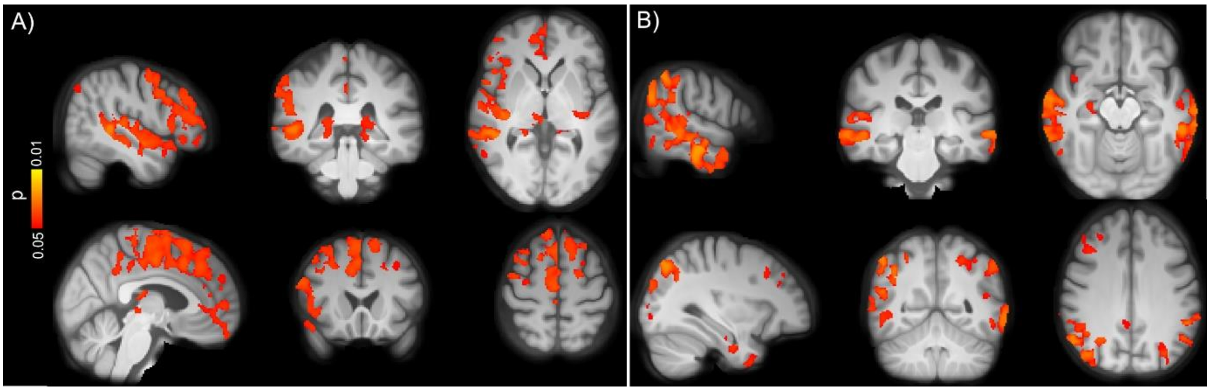

Supplementary Figure 13. Voxel-wise associations between GM-CBF and axonal integrity as measured by CSF concentrations of NfL after additional correction for APOE

ε4 genotype. Higher NfL is accompanied by Lower CBF in both Controls (A) and individuals on the AD spectrum (B) independent of APOE ε4. Conventions as for Figure 3.

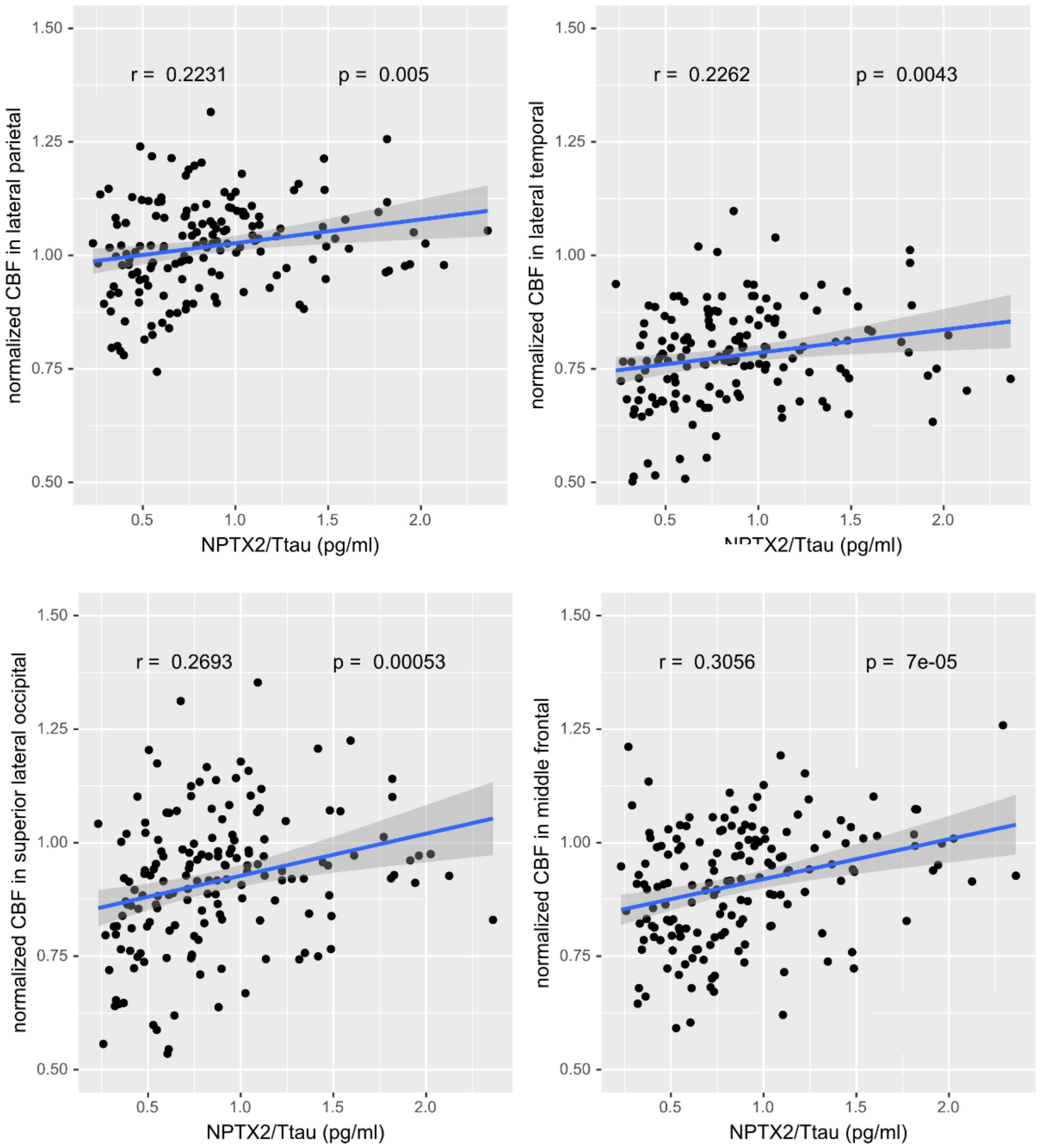

Supplementary Figure 14. ROI-based correlations between CBF and synaptic function. Lower NPTX2/T-tau is associated with hypoperfusion in the AD continuum. Conventions as for Supplementary Figure 8.

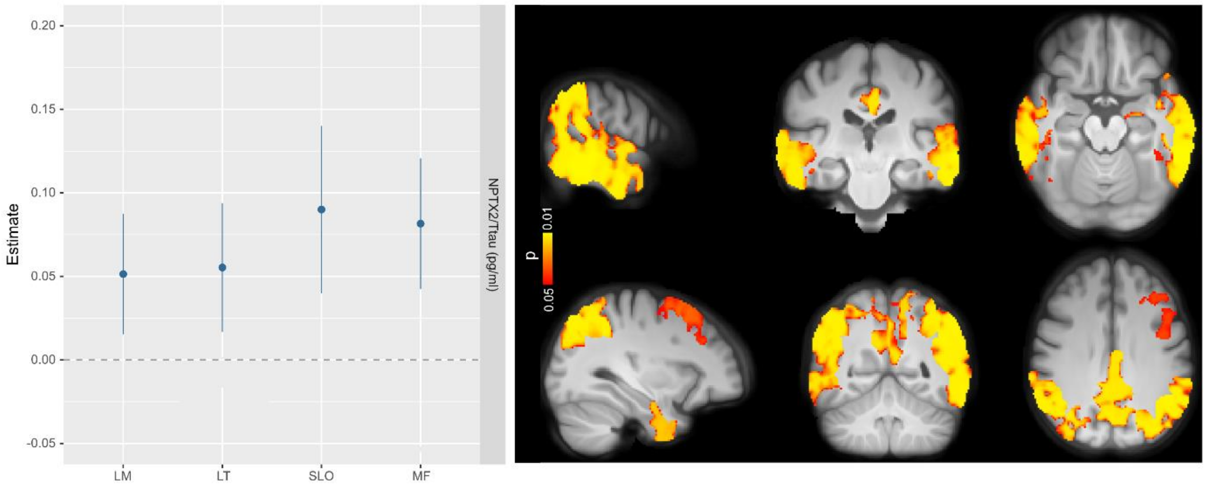

Supplementary Figure 15. ROI-based and voxel-wise associations between CBF and NPTX/T-tau in the AD continuum, corrected for age, sex, ASL sequence version and APOE ε4 genotype. The left panel presents the regression coefficients (β) all of which are significant at $p_{(FDR)} \leq 0.05$, conventions as for Supplementary Figure 6. The voxel-wise associations (right panel) are significant at $p < 0.05$, FWE corrected.

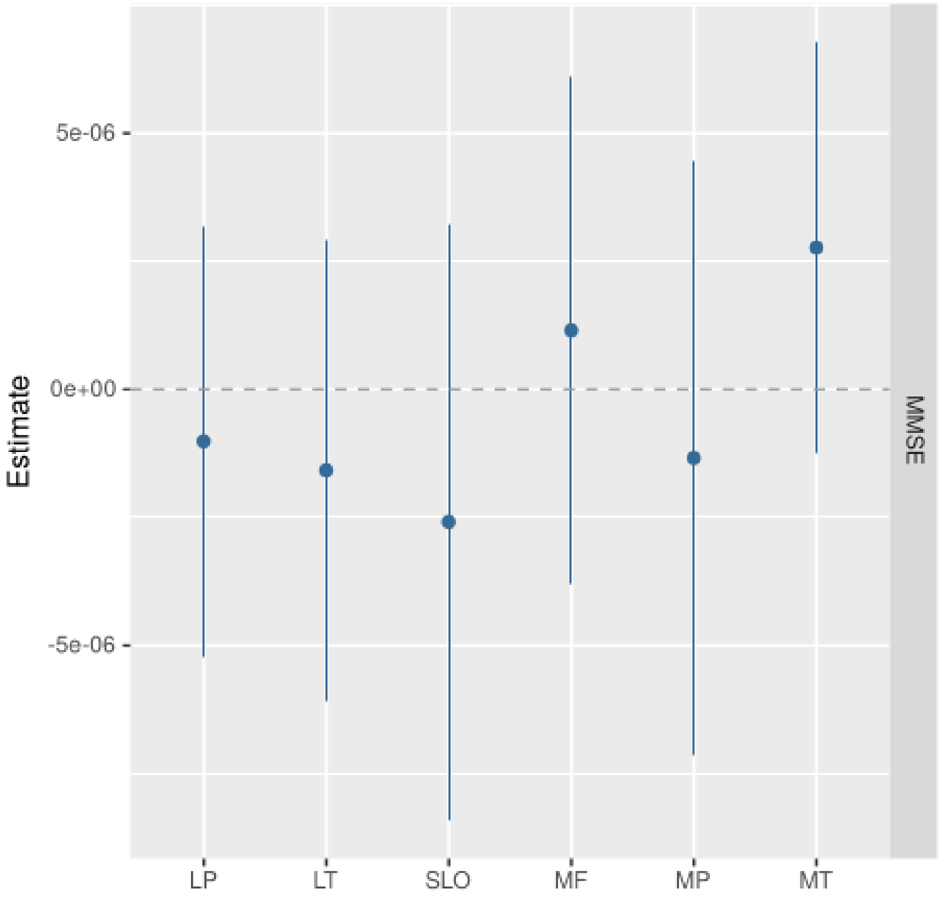

Supplementary Figure 16. Relationship between GM-CBF with global cognitive function in CU individuals, corrected for age, sex, ASL sequence version and education. Given the

highly skewed distribution of MMSE, a Box-Cox transformation was applied to MMSE scores to fulfil the normality assumptions. No ROI-based associations are observed between GM-CSF and MMSE score in this group ($p > 0.05$ uncorrected). Conventions as for Supplementary Figure 6.

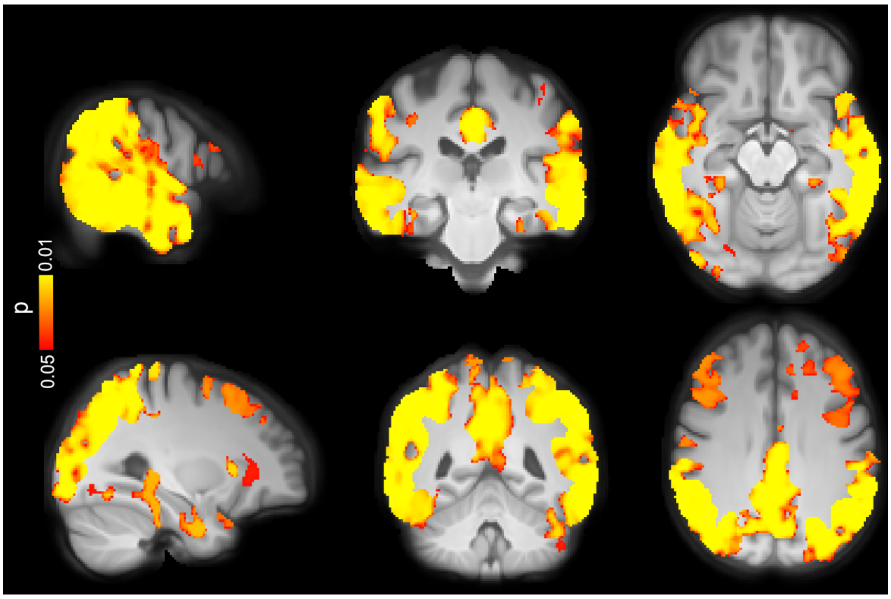

Supplementary Figure 17. Voxel-wise correlations between GM-CBF and global cognition measured by MMSE in the AD spectrum adjusted for age, sex, education and ASL sequence version. Given the highly skewed distribution of MMSE, a Box-Cox transformation was applied to MMSE scores to fulfil the normality assumptions. The main areas with significant associations were located in temporo-occipito-parietal cortex and middle frontal gyrus ($p < 0.05$, FWE corrected). Conventions as for Figure 3.

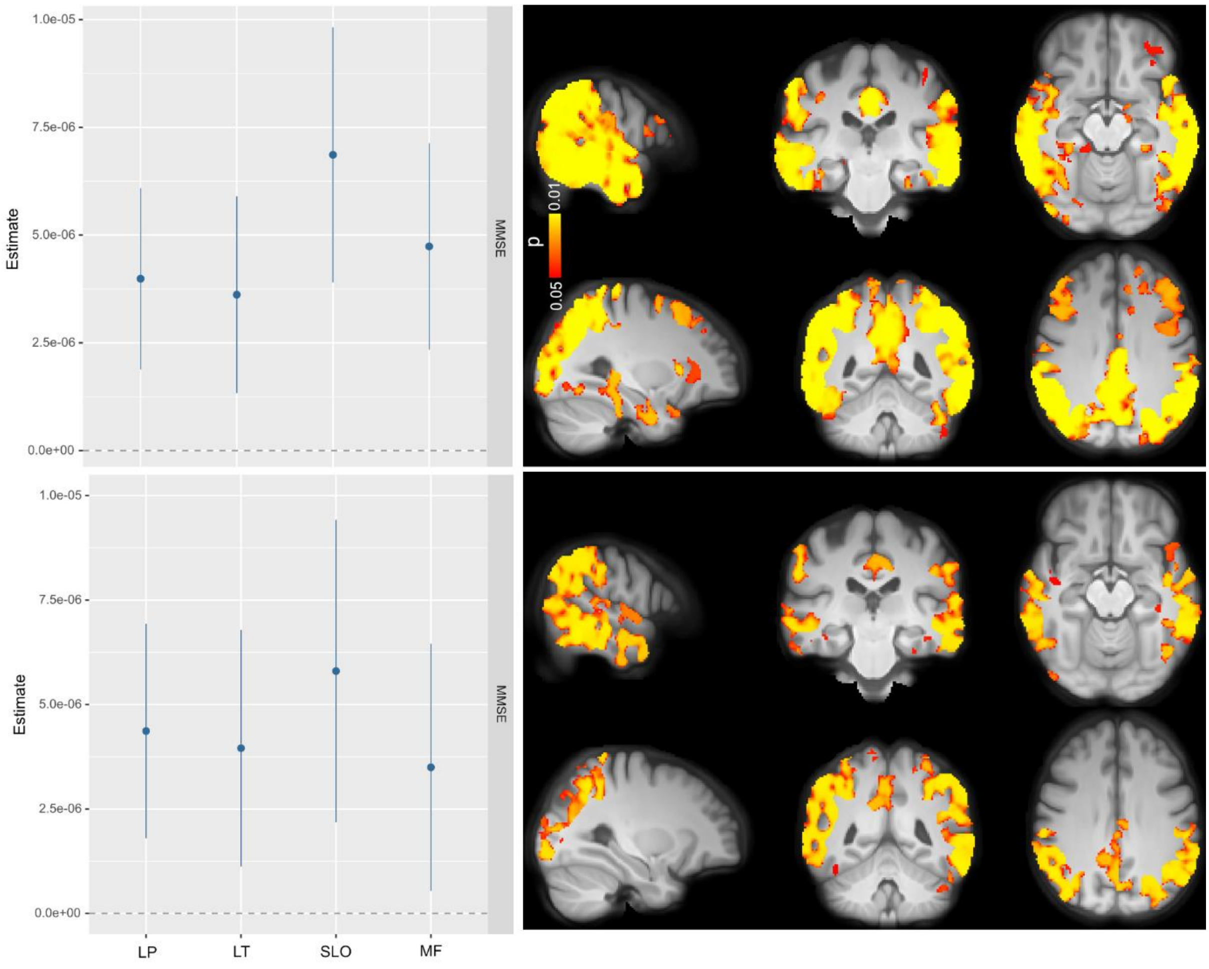

Supplementary Figure 18. ROI-based and voxel-wise associations between GM-CBF and MMSE scores in the AD continuum, corrected for age, sex, education, ASL sequence version, APOE ε4 genotype (top panel) and cognitive status (bottom panel). The left panels display the regression coefficients (β) all of which are significant at $p_{(FDR)} \leq 0.05$, conventions as for Supplementary Figure 6. The right panels represent the voxel-wise associations ($p < 0.05$, FWE corrected). Adjusting for these additional confounding variables does not change the observed positive correlations between hypoperfusion and cognitive deficits.

**sCoV differences between Aβ-negative CU vs Aβ-positive CI**		
**ROI**	**β**	**p (FDR corrected)**
Lateral parietal	0.0606	0.0009***
Lateral temporal	0.0597	0.0001***
Superior lateral occipital	0.0796	0.0004***
Middle frontal	0.0596	0.0014**
Medial parietal	0.0561	0.0014**
Medial temporal	0.0469	0.0014**
**sCoV differences between Aβ-positive CU vs Aβ-positive CI**		
**ROI**	**β**	**p (FDR corrected)**
Lateral parietal	0.0886	0.00009***
Lateral temporal	0.0774	0.00003***

Superior lateral occipital	0.1206	0.00003***
Middle frontal	0.0921	0.00015* **
Medial parietal	0.0785	0.00057***
Medial temporal	0.0715	0.00009***
**sCoV differences between Aβ-negative CU vs Aβ-positive CU**		
**ROI**	**β**	**p (FDR corrected)**
Lateral parietal	-0.0285	0.2091
Lateral temporal	-0.0137	0.3220
Superior lateral occipital	-0.0391	0.2091
Middle frontal	-0.0321	0.2091
Medial parietal	-0.0198	0.3220
Medial temporal	-0.0207	0.2797
**Associations between sCoV and Aβ42/40 in AD spectrum**		
**ROI**	**β**	**p (FDR corrected)**
Lateral parietal	-0.0178	0.8424
Lateral temporal	-0.0403	0.8424
Superior lateral occipital	0.0333	0.8424
Middle frontal	-0.1130	0.7334
Medial parietal	0.0215	0.8424
Medial temporal	0.1720	0.0900
**Associations between sCoV and Aβ42/40 in CU individuals**		
**ROI**	**β**	**p (FDR corrected)**
Lateral parietal	0.0307	0.4909
Lateral temporal	0.0101	0.6622
Superior lateral occipital	0.0456	0.4909
Middle frontal	0.0332	0.4909
Medial parietal	0.0230	0.5605
Medial temporal	0.0429	0.4909
**Associations between sCoV and tau-PET in temporal meta-ROI in AD spectrum**		
**ROI**	**β**	**p (FDR corrected)**
Lateral parietal	0.1042	1.2795e-08***
Lateral temporal	0.0931	5.5737e-10***
Superior lateral occipital	0.1356	1.4280e-09***
Middle frontal	0.0949	1.9200e-06***
Medial parietal	0.0867	1.9200e-06***
Medial temporal	0.0517	2.7540e-04***
**Associations between sCoV and tau-PET in temporal meta-ROI in CU individuals**		
**ROI**	**β**	**p (FDR corrected)**
Lateral parietal	0.0312	0.6796
Lateral temporal	0.0423	0.5764
Superior lateral occipital	0.1168	0.3395
Middle frontal	0.0647	0.5764
Medial parietal	0.0306	0.6796
Medial temporal	0.0063	0.8879
**Associations between sCoV and NPTX2/T-tau in AD spectrum**		
**ROI**	**β**	**p (FDR corrected)**
Lateral parietal	-0.0987	7.2559e-05***

Lateral temporal	-0.0929	4.5451e-06***
Superior lateral occipital	-0.1450	4.5451e-06***
Middle frontal	-0.1077	7.2559e-05***
Medial parietal	-0.0595	0.01837*
Medial temporal	-0.0560	0.00496**
**Associations between sCoV and NPTX2/T-tau in CU individuals**		
**ROI**	**β**	**p (FDR corrected)**
Lateral parietal	-0.0178	0.3464
Lateral temporal	-0.0167	0.3265
Superior lateral occipital	-0.0296	0.3265
Middle frontal	-0.0301	0.3265
Medial parietal	-0.0042	0.9566
Medial temporal	-0.0001	0.9892

Supplementary Table 11. Sensitivity ROI-based analysis on between-group differences in spatial coefficient of variation (sCoV) and its association with markers of amyloid and tau pathology as well as synaptic dysfunction. As sCoV data were not normally distributed, linear regressions were performed with its log-transformed values. Age, sex, ASL sequence version, a combined score for diseases including congestive heart failure, hyperlipidemia, arterial fibrillation, ischemic heart disease, diabetes, and hypertension as well as a combined score for medications (lipid-lowering, platelet-inhibitors, cardioprotective pills) were included in the model as nuisance covariates. Except for the medial parietal and medial temporal ROIs, these results are consistent with the observed findings when using GM-CBF as the main outcome variable indicating that the reported results are largely independent of the vascular burden.
